# Cognitively impaired aged *Octodon degus* recapitulate major neuropathological features of sporadic Alzheimer’s disease

**DOI:** 10.1186/s40478-022-01481-x

**Published:** 2022-12-19

**Authors:** Zhiqun Tan, B. Maximiliano Garduño, Pedro Fernández Aburto, Lujia Chen, Nicole Ha, Patricia Cogram, Todd C. Holmes, Xiangmin Xu

**Affiliations:** 1 Department Anatomy and Neurobiology, School of Medicine, University of California, Irvine, CA 92697 USA; 2 Institute for Memory Impairments and Neurological Disorders, University of California, Irvine, CA 92697 USA; 3 Institute of Ecology and Biodiversity, Department of Ecological Sciences, Faculty of Sciences, University of Chile, Santiago, Chile; 4 Department Physiology and Biophysics, School of Medicine, University of California, Irvine, CA 92697 USA; 5 The Center for Neural Circuit Mapping, University of California, Irvine, CA 92697 USA

**Keywords:** Degu, Natural model, Outbred, Behavioral, Immunostaining, Comprehensive investigation

## Abstract

The long-lived Chilean rodent (*Octodon degus*) has been reported to show spontaneous age-dependent neuropathology and cognitive impairments similar to those observed in human AD. However, the handful of published papers on degus of differing genetic backgrounds yield inconsistent findings about sporadic AD-like pathological features, with notably differing results between lab in-bred degus versus outbred degus. This motivates more extensive characterization of spontaneously occurring AD-like pathology and behavior in degus. In the present study, we show AD-like neuropathological markers in the form of amyloid deposits and tau abnormalities in a cognitively impaired subset of aged outbred degus. Compared to the aged degus that show normal burrowing behavior, the age-matched degus with burrowing behavior deficits correlatively exhibit detectable human AD-like Aβ deposits and tau neuropathology, along with neuroinflammatory markers that include enhanced microglial activation and higher numbers of reactive astrocytes in the brain. This subset of cognitively impaired aged degus also exhibits cerebral amyloid angiopathy and tauopathy. We find robust neurodegenerative features in behaviorally deficient aged degus, including hippocampal neuronal loss, altered parvalbumin and perineuronal net staining in the cortex, and increased c-Fos neuronal activation in the cortex that is consistent with the neural circuit hyperactivity reported in human AD patients. By focusing on the subset of aged degus that show AD-like behavioral deficits and correlative neuropathology, our findings establish outbred degus as a natural model of sporadic AD and demonstrate the potential importance of wild-type outbred genetic backgrounds for AD pathogenesis.

## Introduction

Alzheimer’s disease (AD) is an age-related progressive neurodegenerative disorder characterized by irreversible cognitive decline and specific pathologic lesions in the brain that greatly impair the lives of individuals suffering from the condition. There are approximately 44 million people suffering from AD worldwide, of which over 90% of those cases are late-onset and occur sporadically [1]. As of today, more than 170 different genetically modified or pharmacologically induced animal mouse models have been developed for the study of this brain-disruptive neurological disorder [2, 3]. Although mouse models exhibiting certain Alzheimer’s features have furthered our understanding of AD, short lived mouse models do not fully recapitulate the important characteristics of human AD, particularly those seen in sporadic late-onset cases. The neuroanatomical distribution of AD neuropathologies, such as beta-amyloid (Aβ) and neurofibrillary tangles (NFT), show distinct hippocampal to cortical spatio-temporal gradients that intensify with disease progression [4]. In addition to progressive cognitive deficits and accumulation of the typical pathologic hallmarks of Aβ plaques and NFTs, human AD brains exhibit additional neuropathological features including significant neuronal loss, neuroinflammation, widespread white matter abnormalities, and selective vasculature lesions [5, 6]. There is an urgent need to find natural Alzheimer’s models that better recapitulate all the behavioral abnormalities and pathological features of late onset AD.

Degus (*Octodon degus*) are rodents native to Chile characterized by their diurnal activity, highly social nature, and long lifespan (up to 8 years of age) with measurable cognitive capabilities. They are known to develop behavioral and memory deficits with the progression of age [7]. The high Aβ amino acid homology (harboring only one residue difference, i.e., H13R) between degus and humans confers a propensity for degu Aβ to form AD-like aggregates more readily than mouse Aβ [8], as both mouse and rat Aβ42 (which are identical to each other) have three-residue differences (R5G, Y10F, and H13R) compared to human Aβ42. Outbred degus can spontaneously develop age-related cognitive dysfunction. Thus, degus may be a promising natural model candidate of AD [9, 10]. However, previous investigation has been relatively preliminary, and different studies using different strains of degus have reported inconsistent observations of no measurable Alzheimer’s-related pathologies, most notably in the brains of lab in-bred degu strains [11, 12].

To resolve these inconsistencies and to determine whether degus are a good natural model of sporadic AD, we evaluated neuropathologic features in the brains of outbred degus in combination with behavioral testing. Results revealed a subset of aged outbred degus that exhibit cognitive behavioral deficits. These cognitively impaired degus also show AD-like neuropathological features, while age-matched degus that are not behaviorally impaired do not exhibit these same neuropathological features. In addition to measurable Aβ plaques and neuroinflammation, we detect a significant neuronal loss and buildup of phosphorylated tau-related NFT-like deposits in hippocampus, entorhinal cortex, retrosplenial cortex, and white matter areas only in the cognitively impaired aged degus. The neuropathological features in cognitively impaired degus and their neural-circuit/spatial distribution are similar to those often observed in the postmortem human brain with late-onset sporadic AD.

## Materials and methods

### Animals, burrowing behavior testing and tissue processing

All experimental procedures were conducted under protocols approved by the ethics committee of the Faculty of Sciences of the University of Chile and the University of California Irvine Institutional Animal Care and Use Committee.

**Fig. 1:**
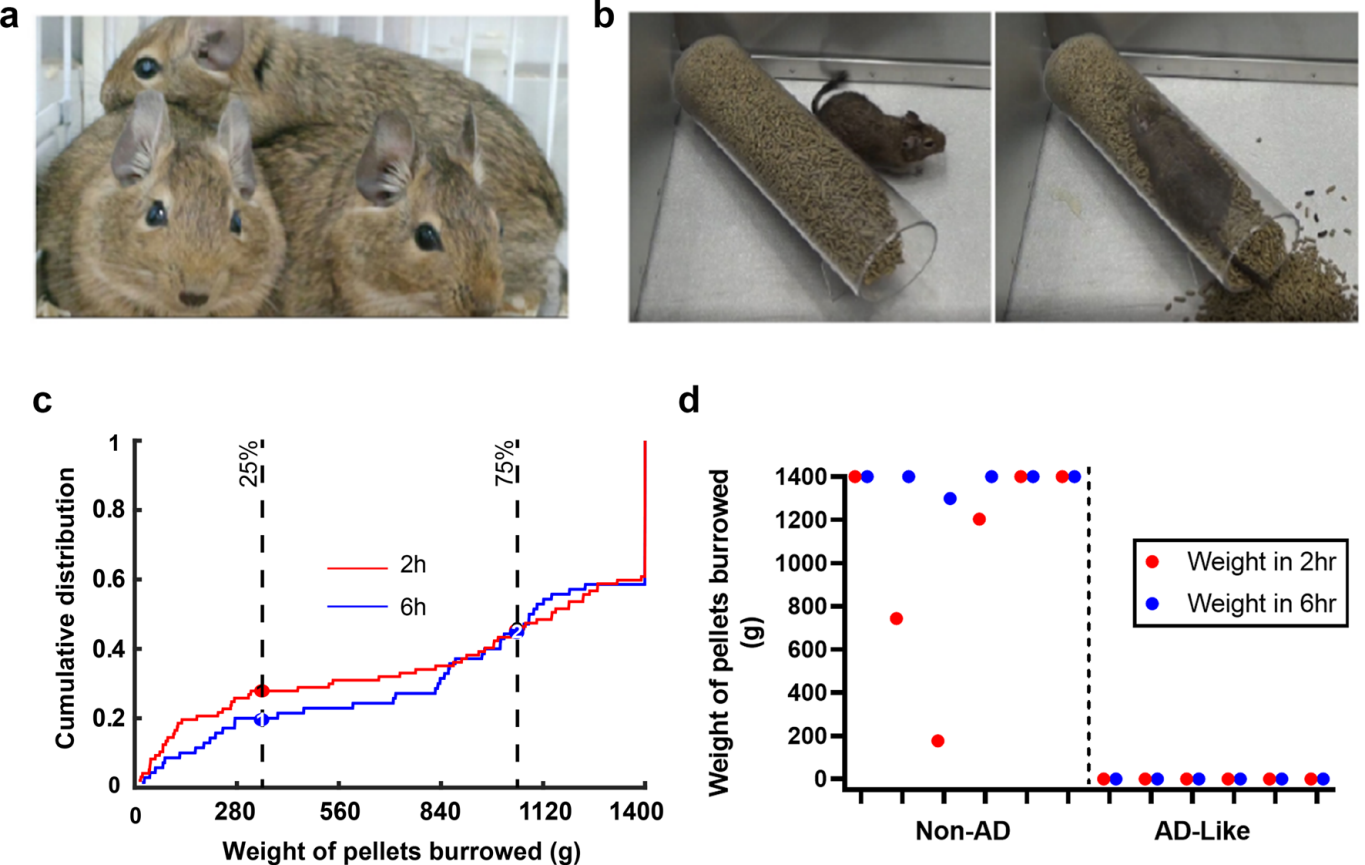
Degus behavioral burrowing performance as a screen for normal versus AD-like aged degus. **a** A photo of outbred degus in the colony directed by Dr. P. Cogram. **b** Representative images extracted from a video recorded during the degu performance of the burrowing test. **c** The cumulative distributions of the weights of pellets burrowed in 2 h (red curve) and in 6 h (blue curve). The (1) and (2) circles indicate the points of 0.25 and 0.75 cumulative data points for the 2-hour curve; the (1) and (2) circles indicate the points of 0.25 and 0.75 cumulative data points for the 6-hour curve (n = 146 degus, 4.5–5 years old). **d** Scatter plot showing the distribution of pellets burrowed after 2 h (red dots) and 6 h (blue dots) in the 6 age matched control degus and the 6 AD-like degus, all of which were neuropathologically examined in this study

All degus (*Octodon degus*) used in the present study were from a genetically diverse degu colony stocked using wild caught animals at the Institute of Ecology and Biodiversity, University of Chile, Santiago, Chile. Wild caught pregnant females had their outbred pups in the colony. Once weaned from mother, each outbred degu was labelled with a unique ID number and lived in the colony for its entire life. The colony maintains an outbred degu population. Degus were housed in 50 cm x 40 cm x 35 cm metal cages with wood shaving beddings at 23 °C in same sex groups of up to 4 animals (due to the social nature of degus). Water and food (rodent RMH 3000) were available *ad libitum* to the animals. At ages ranging from 4 to 5.5 years (all spent in the colony), 146 degus were subject to a burrowing behavior test, which allows for a sensitive evaluation of an ethologically-relevant cognitive performance evaluation in rodents [13, 14]. A full burrow (a 200 mm long, 68 mm diameter tube filled with 1400 g of pellets) was placed into an individual test cage (Deacon, 2006). The amounts of pellets burrowed from these tubes were measured after 2 and 6 h. Across three 48-hour apart burrowing tests, degus that burrowed less than 25% of the pellets (350 g) after 6 h were assigned to the “AD-like”, *poor burrower* group, while those that burrowed more than 75% of the pellets (1050 g) were assigned to the “non-AD”, *good burrower* group (Fig. 1).

Upon completion of the behavioral test, 12 selected degus ranging from 4 to 5.5 years of age (6 AD-like and 6 non-AD, 3 males and females in each group as listed in Table 1) were euthanized for brain collection. Dissected brains (right hemisphere) were fixed in 4% paraformaldehyde (PFA) in 1x phosphate buffer saline (PBS, pH 7.4) for 24 h at 4 °C, then soaked in 30% sucrose in PBS for 3 days prior to sectioning. Brain coronal sections at slice thickness of 30 μm were prepared on a Leica SM2010 sliding microtome. Serial free-floating coronal sections were harvested in 1xPBS and subsequently transferred to cryoprotective solution for − 20 °C storage prior to staining.

**Table 1:** Individual degu information

ID	Age (months)	Sex	Classification
181	60	Female	Non-AD
309	56	Female	Non-AD
473	68	Female	Non-AD
336	68	Male	Non-AD
392	56	Male	Non-AD
3816	63	Male	Non-AD
316	66	Female	AD-like
3680	66	Female	AD-like
6998	65	Female	AD-like
3848	65	Male	AD-like
6204	60	Male	AD-like
6349	66	Male	AD-like

Both 5xFAD (7-month-old) and 3xTg-AD (14-month-old) mice used in this study were obtained from the UCI MODEL-AD Consortium. Mice were euthanized and transcardially perfused with PBS followed by 4% PFA in 1xPBS for fixation. Mouse brains were then harvested, further fixed in 4% PFA overnight at 4 °C, and processed using the same protocol used for the degu brains. Human postmortem frontotemporal cortex (10% formalin fixed) was kindly provided by USC ADRC Dr. Carol Miller’s laboratory. Microtome sections were prepared as above.

### Immunochemical staining

Sections were labeled using the following immunohistochemistry protocol. Briefly, for immunofluorescence staining, free-floating coronal sections were rinsed with 1x phosphate buffered saline (PBS), incubated in blocking buffer containing 5% normal donkey serum (Jackson ImmunoResearch Laboratories, West Grove, PA; #017-000-121) and 0.1% (v/v) Triton X-100 in 1xPBS followed by lipofuscin autofluorescence blocking using TrueBlack (Biotium, Fremont, CA. #23007) according to the manufacturer’s protocol. Next, sections were sequentially incubated with primary antibodies (including 6E10, Aß40, Aß42, Aß43, pyroGlu3Aß (AßpE3), mOC23, mOC31, ubiquitin, smooth muscle α-actin (SMA) NeuN, PV, GFAP, IBA-1 and c-Fos) using proper dilutions (Table 2) for 72 h at 4 °C, washed in 1xPBS, incubated with appropriate fluorescent secondary antibody diluted in 1xPBS (as detailed in Table 2) containing 4’,6-diamidino2phenylindole (DAPI) (ThermoFisher; D1306, 10µM,) for 30 min at room temperature, and washed in 1xPBS. Finally sections were mounted and coverslipped with Fluoromount-G (SouthernBiotech, Birmingham, AL; #0100-01) for microscopic imaging.

**Table 2:** Reagents and resources with Research Resource Identifiers (RRID) tags

Reagent or resource	Source	Identifier	City & State	RRID	Dilution
*Antibodies*					
6E10 beta-Amyloid 1–16(mouse monoclonal)	BioLegend	803001	San Diego, CA, USA	*AB_2564653*	1:500
Anti-Abeta 40 (beta amyloid) (rabbit polyclonal)	MyBiosource	MBS555166	San Diego, CA, USA	*AB_2920623*	1:500
Anti-Abeta 42 (beta amyloid) (mouse monoclonal)	MyBiosource	MBS592140	San Diego, CA, USA	*AB_2920624*	1:500
Anti-Abeta 43 (beta amyloid) (mouse monoclonal)	BioLegend	805601	San Diego, CA, USA	*AB_2564686*	1:500
Anti-beta amyloid [Pyro Glu3] (polyclonal)	Novus	NBP1-44048	San Diego, CA, USA	*AB_2056697*	1:500
Anti- beta amyloid mOC23 (rabbit monoclonal)	Abcam	ab205340	Waltham,MA, USA	*AB_2920639*	1:500
Anti- beta amyloid mOC31 (rabbit monoclonal)	Abcam	ab205340	Waltham,MA, USA	*AB_2920640*	1:500
Phospho-Tau (Ser202, Thr205) AT8 (mouse monoclonal)	Thermo fisher scientific	MN1020	Waltham, MA, USA	*AB_223647*	1:500
Human-Tau40 159PPGQK163HT7 (mouse monoclonal)	Thermo fisher scientific	MN1000	Waltham, MA, USA	*AB_223647*	1:500
Human Tau40 306VQIVYK311 amyloid motif (rabbit polyclonal)	ABclonal	A19560	Woburn, MA, USA	*AB_2862667*	1:250
Anti-PHF (Ser396, Ser404)	P. Davies. Albert Einstein College of Medicine	PHF1	NY, USA	*AB_2315150*	1:500
Anti-GFAP (chicken polyclonal)	Abcam	ab4674	Waltham,MA, USA	*AB_304558*	1:1000
Anti-Iba1 (rabbit polyclonal)	FUJIFILM WAKO	019-19741	Richmond, VA, USA	*AB_839504*	1:1000
Anti-Parvalbumin (rabbit polyclonal)	Swant	PV-28	Burgdorf, CHE	*AB_2315235*	1:1000
Anti-NeuN (mouse monoclonal)	Abcam	ab104224	Waltham,MA, USA	*AB_10711040*	1:1000
Anti-cFos (rabbit polyclonal)	Abcam	ab190289	Waltham,MA, USA	*AB_2737414*	1:500
Anti-ubiquitin, P4D1 (mouse monoclonal)	Santa Cruz biotechnology	sc-8017	Dallas, TX, USA	*AB_628423*	1:250
Anti-α-smooth muscle actin (mouse monoclonal)	Santa Cruz biotechnology	sc-53,142	Dallas, TX, USA	*AB_2273670*	1:250
Cy3 Donkey anti mouse (secondary, polyclonal)	Jackson immuno research	715-165-150	West Grove, PA, USA	*AB_2340813*	1:200
Cy5 Donkey anti mouse (secondary, polyclonal)	Jackson immuno research	715-175-150	West Grove, PA, USA	*AB_2340819*	1:200
Cy3 Donkey anti rabbit (secondary, polyclonal)	Jackson immuno research	711-165-152	West Grove, PA, USA	*AB_2307443*	1:200
Alexa Fluor 488 Donkey anti rabbit (secondary, polyclonal)	Jackson immuno research	711-545-152	West Grove, PA, USA	*AB_2313584*	1:200
Cy3 Donkey anti Goat (secondary, polyclonal)	Jackson immuno research	705-165-147	West Grove, PA, USA	*AB_2307351*	1:200
Alexa Fluor 488 Donkey anti Goat (secondary, polyclonal)	Jackson immuno research	705-545-147	West Grove, PA, USA	*AB_2336933*	1:200
Alexa Fluor 488 Donkey anti Chicken (secondary, polyclonal)	Jackson immuno research	703-545-155	West Grove, PA, USA	*AB_2340375*	1:200
*Chemicals, stains and kits*					
Amylo-Glo RTD	Biosensis	TR-300-AG	Thebarton, AUS		1:100
Wisteria Floribunda Lectin/Agglutinin (WFA, WFL), Biotinylated	Vector laboratories	B-1355-2	Newark, CA, USA		1:1000
Abeta40 ELISA kit	MyBioSource	MBS760432	San Diego, CA, USA		
Abeta42 ELISA kit	MyBioSource	MBS726927	San Diego, CA, USA		
Alexa Fluor 488 Streptavidin	Jackson Immuno Research	016-540-084	West Grove, PA, USA		1:500
TrueBlack lipofusion autofluorescence quencher	Biotium	23007	Fremont, CA		1:20

To stain amyloid plaques with Amylo-Glo, free-floating coronal sections were incubated in 70% ethanol followed by a brief wash in distilled water. The sections were then stained with Amylo-Glo RTD Amyloid Plaque Stain Reagent (Biosensis, cat# TR-300-AG, 1:100 dilution) in 0.9% saline for 10 min at room temperature per manufacturer’s instruction. Then, sections were washed in 0.9% saline and rinsed briefly in distilled water followed by blocking in 5% donkey serum and 0.1% (v/v) Triton X-100 in 1xPBS for 30 min followed by the immunofluorescence protocol described above.

Perineuronal nets (PNN) were immunostained using biotinylated Wisteria floribunda agglutinin (WFA; Vector Laboratories, cat# B-1355-2). WFA is a lectin, and referentially binds to glycosaminoglycan side chains of chondroitin sulfate proteoglycans found in PNNs [15]. Initially, free floating slices were rinsed three times with 1xPBS and followed by incubation in blocking solution containing 5% normal donkey serum and 0.075% Triton X in 1xPBS for 2 h. This was followed by incubation in biotinylated Wisteria floribunda lectin (WFA, Vector Laboratories, Burlingame, CA; 1:1000) in blocking solution) for 72 h at 4 °C. Sections were then rinsed in 1xPBS followed by incubation in Alexa Fluor 488 conjugated streptavidin (1:500) in 5% blocking solution in 1xPBS for 2 h, PBS washing, and counterstained with 10 µM DAPI, then finally mounted and coverslipped with Fluoromount-G for confocal microscopy.

### Brain section imaging

To obtain slice image overviews, slices coverslipped on microscope slides were imaged using a high-throughput Olympus VS120 scanning system. Confocal microscopy was conducted (Leica SP8 or Olympus FV3000) to obtain high resolution images, using a series of optical sections that were captured for each slice using a 10x or 20x objective, with 1x or 2.5x zoom at 1 μm step interval for 10–12 slices (z-stack). The maximum intensity projection 2D images were processed by iMaris software (Version 9.7.0, Oxford Instruments). All images were acquired under identical conditions.

### ELISA measurements of Aßx-40 and Aßx-42 in degu brain tissues

To further confirm Aß accumulation in the degu brains, five 30 μm hippocampus-containing coronal slices from each brain were picked for preparation of formic acid-extracted brain homogenates in 320 ml formic acid-containing solution as described [16–18]. Supernatants were collected after centrifugation (20,000 g x 30 min, 4 °C) and 100 ml neutralized supernatant was applied to each ELISA assay. ELISA analysis was conducted using the quantitative sandwich ELISA kits for human Amyloid Beta 40 and Amyloid Beta 42 (MyBioSource, San Diego, CA). All measurements were triplicated for each sample and performed according to the manufacturer’s protocol.

### Image quantification and analysis

Analysis of neuroinflammation markers using Imaris: z-stack images obtained from Leica SP8 were deconvoluted using the wizard function of Huygens Essential (Scientific Volume Imaging, Netherlands) followed by Imaris software (Version 9.7.0, Oxford Instruments). Imaris was used to reconstruct the astrocytic and microglial surface to measure cell volume. Settings for surface analysis of astrocytic reconstruction are as follows: surfaces detail 0.4 μm (smooth); thresholding background subtraction (local contrast), diameter of largest sphere, which fits into the object: 18 μm; threshold (background subtraction): 20, filter “Quality”: 3.5. The surface analysis settings for microglial surface reconstruction are as follows: surfaces detail 0.5 μm (smooth); thresholding background subtraction (local contrast), diameter of largest sphere, which fits into the object: 10 μm; threshold (background subtraction): 15, filter “Quality”: 3.5. After surface reconstruction, all astrocytes and microglia with incomplete somata due to cutting, were manually removed and not included in further analysis. Astrocytic and microglial processes were further analyzed using Imaris filament analysis. Seed points were manually corrected if Imaris automatic tracing algorithm place points incorrectly. Additionally, filaments not attached to soma were manually removed. All surface and filament analysis were exported into Excel files and used for data analysis.

Sholl analysis was performed to demonstrate the complexity of microglial processes for the activation status using Imaris filament reconstruction analysis. The center of all concentric spheres was defined as the center of the microglial soma. The starting radius from center of the soma was 5 μm and the ending radius was 60 μm with an interval of 5 μm between radial intersections.

PNN, PV, c-Fos, NeuN, and vascular Aß immunostaining signal was analyzed in all 12 degus (n = 6 for AD-like, n = 6 for Non-AD). Regions of interest were selected based on anatomical brain regions known to be impacted in Alzheimer’s disease. The degu brain atlas was used on degu coronal sections to locate these regions of interest and analyze them appropriately [19]. Maximum projection images covering randomly selected singular or multiple ROIs from confocal images of one section per animal were used to measure the intensity of specific immunoreactivity signals and subsequently analyzed using ImageJ software. PNN and PV positive cells per mm^2^ were assessed in entorhinal cortex (EC), hippocampal CA3/CA2, and thalamic reticular nucleus using 1 ROI per degu sample (of sizes 0.4096 mm^2^, 1.6384 mm^2^, and 10.02 mm^2^ respectively for each). Cells per mm^2^ were manually counted in EC (PNN and PV) and hippocampal CA3/CA2 (PV), while ImageJ analysis using a threshold to remove background followed by particle analysis was used to determine total signal in hippocampal CA3/CA2 (PNN) and thalamic reticular nucleus (TRN) (PNN and PV). This signal was subsequently divided by the typical area of a PNN or PV cell to determine cells per mm^2^. Relative intensity was calculated for c-Fos signal in EC, RSC, and hippocampal CA1 (all 0.4096 mm^2^ ROI’s) by measuring mean intensity values and subtracting background signal in each image (4 ROIs per degu sample). Neurons (NeuN) per mm^2^ were manually counted in distal hippocampal CA1 (3 ROI’s per degu section) with each ROI of 0.0668 mm^2^. Vascular deposition of Aß was identified by dual fluorescent labeling of Aßx-40 and α-smooth muscle actin (SMA) and manually count ed as the percentage of both Aßx-40 and SMA co-labeled vessels over all SMA-positive vascular structures in 1.69 mm^2^ ROI’s (a 10x confocal image), and 3 ROI’s for each anatomical area (hippocampus, somatosensory cortex (S1) and thalamus) were examined for each degu.

6E10 signal analysis: Conventional epifluorescence images of degu somatosensory cortex (S1) and dorsal hippocampus (dHIP) were analyzed with Photoshop for immunoreactive signal areas (n = 6 for each degu group, 1 ROI per area). Plaque deposits were manually counted in regions of interest and converted to counts per µm^2^.

### Statistical analyses

Statistical analyses were performed using GraphPad Prism 7 (GraphPad Software, CA, USA) or Matlab custom scripts. Statistical analysis of burrowing performance, ELISA measurements, astrocytic and microglial parameters used individual measurement or cell values from Non-AD versus AD-like degu groups. The generation and statistical comparison of Sholl distribution curves used individual microglial values per non-AD and AD-like degu groups. Data were presented as mean ± standard error of the mean (SEM). Statistical results were considered significant if p < 0.05 and are presented as * for p < 0.05, ** for p < 0.01, *** for p < 0.001, and **** for p < 0.0001 in the respective figures. Non-parametric Mann-Whitney *U*-tests were used for data comparisons between Non-AD and AD-like groups of degus when appropriate, whereas the linear mixed-effect model (LME) was applied to address the issue of correlated data due to repeated measurements from multiple single animal brain samples. The main idea of LME is that the data outcome is affected by fixed effects, which can be considered as the conditions we want to test, as well as random effects are considered as the potential groupings of data that constitute repeated measurements, such as the animals from which the groups of neurons are repeatedly measured or collected (Yu et al., 2021). When conducting statistical modeling and hypothesis testing, the influence of both fixed and random effects are taken into account, which handles the potential artificial increase of statistical power by multiple dependent measurements [20, 21]. In this paper, the LME analysis was performed using “fitlme” function provided by MATLAB. The detailed information for the quantification and analysis of the immunofluorescence markers assayed in the current study is summarized in Table 3.

**Table 3:** Methods use for immunofluorescence marker quantification and analysis

Marker	Areas assessed	ROIs per area	Statistics used for analysis
6E10 (Aβ)	Somatosensory cortex Dorsal hippocampus	1	Mann-Whitney test
Aβ40-SMA/SMA (Vascular Aβ)	Somatosensory cortex Hippocampus Thalamus	3–4	Linear mixed-effect model (LME)
GFAP (Reactive astrocytes)	Somatosensory Cortex Hippocampus	3	Linear mixed-effect model (LME)
Iba-1 (Activated microglia)	Somatosensory Cortex Hippocampus	3	Linear mixed-effect model (LME)
NeuN (Neuronal cell loss)	Hippocampal distal CA1	3	Linear mixed-effect model (LME)
c-Fos (Cell activity)	Entorhinal cortex Retrosplenial cortex Hippocampal CA1	4	Linear mixed-effect model (LME)
WFA (Perineuronal nets)	Entorhinal cortex Hippocampal CA3/CA2 Thalamic reticular nucleus	1	Mann-Whitney test
PV (Parvalbumin positive cells)	Entorhinal cortex Hippocampal CA3/CA2 Thalamic reticular nucleus	1	Mann-Whitney test

## Results

### Behavioral burrowing deficits in a subpopulation of aged degus

Digging behavior is an innate behavior that integrates hippocampal and motor function in degus. Behavioral deficits in food burrowing performance occur following site directed lesions in the hippocampus and prefrontal cortex in rodents as well as degus [14, 22, 23]. We tested 146 degus between the ages of 4.5 to 5.5 years using a burrowing assay to identify degus with ethologically relevant cognitive impairments (Fig. 1). A subset of degus exhibit no burrowing activity (i.e., 0 g of pellets were burrowed) in either of the 2-hour or 6-hour test periods, whereas about 40% of the tested degus finished burrowing all of the 1400 g of pellets within 6-hours (Fig. 1c). Young healthy degus show no deficits in burrowing activity [14]. Degus demonstrating the lowest 25% and top 75% burrowing performance within the 6 h test period were grouped as “AD-like” and “Non-AD” degus, respectively. The burrowing performance of twelve degus (non-AD-like versus AD-like) is shown in Fig. 1d, and their brains were subsequently analyzed for the presence or absence of neuropathological features.

### Significantly higher Aß brain deposits correlate with behavioral deficits in burrowing performance in aged degus

**Fig. 2:**
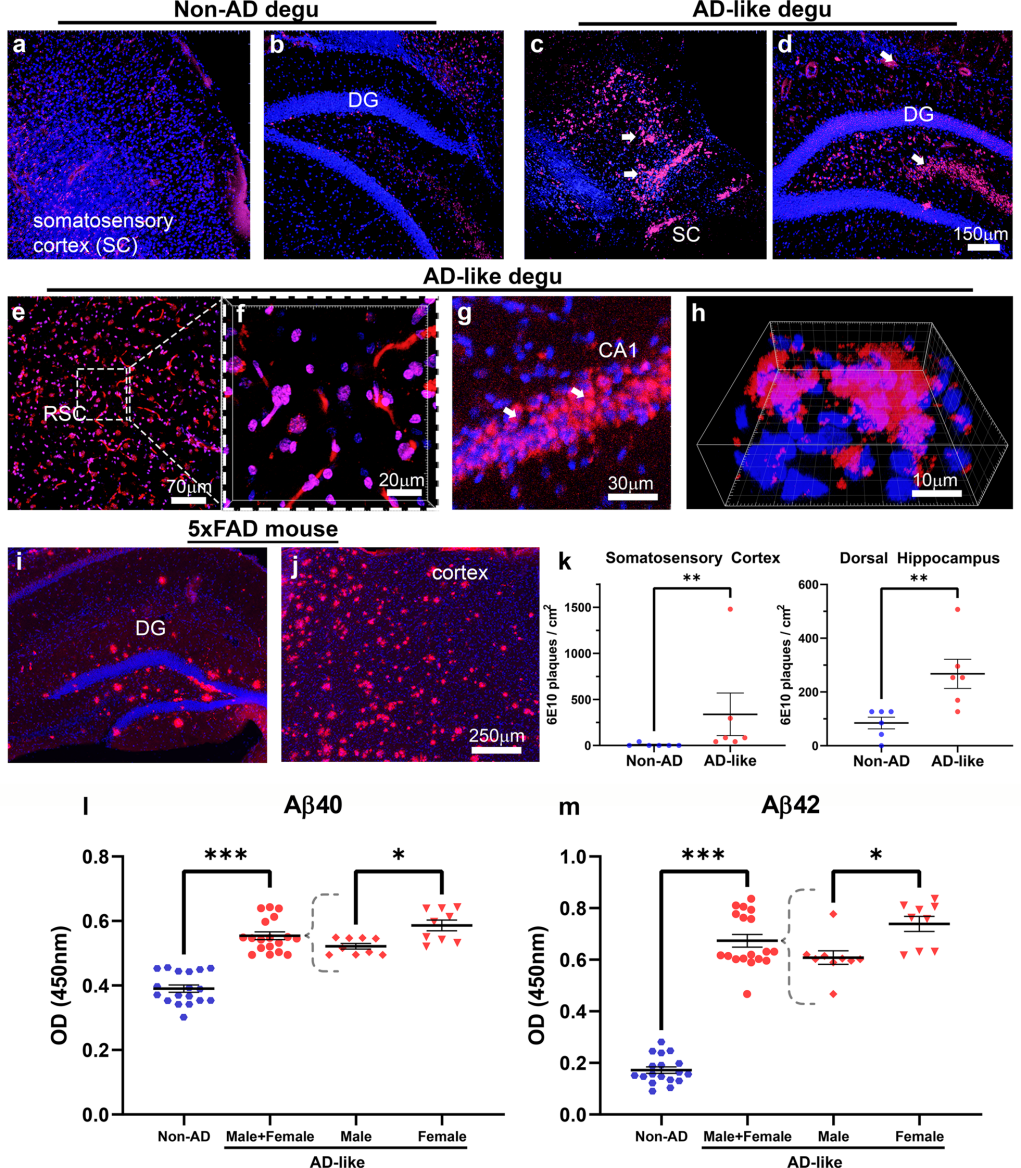
AD-like aged degu brains show greater Aβ deposits relative to age matched controls. (**a**–**h**) Confocal fluorescence micrographs demonstrate markedly more Aβ deposits (red) in degu coronal brain slices stained with 6E10 monoclonal antibody anti-Aβ(1–16) N-terminal epitope. Representative views of cortex including somatosensory cortex (SSC) and hippocampus dentate gyrus (DG) are from Non-AD (**a**, **b**) and AD-like aged degu (**c **and **d**, arrows indicate 6E10-positive Aβ deposits) brains. Images highlight the massive Aβ neuritic-plaque-like deposits in the retrosplenial cortex (RSC, **e**, **f**), cytoplasmic deposition in hippocampal CA1 (**g**, arrows indicate 6E10-positive Aβ deposits), and a Z-stack 3D reconstruction view of a large plaque in a cortical area in AD-like aged degus (**h**). **i**, **j** Representative immunofluorescence microscopic views covering hippocampal DG and a cortex area of coronal slices of an aged 5xFAD brain stained same as the degu sections. All sections were counterstained with DAPI for nuclei as blue. Note that overlap in DAPI (blue) and 6E10 (red) signal results in magenta color. **k** The 6E10-positive deposits per cm^2^ is quantified in AD-like and Non-AD aged degus (*N* = 6 for each degu group) in the SC and dorsal hippocampus (dHIP). Results show statistical significance for the 6E10 + plaques per cm^2^ between AD-like and Non-AD groups. **p < 0.01. (**l**, **m**) Analysis of Aβ40 and Aβ42 in formic acid extracts from Non-AD and AD-like degu brain slices by ELISA demonstrates increased levels of Aβ40 and Aβ42 in AD-like compared to Non-AD degu brains; whereas AD-like females show higher levels than AD-like males (N = 3 for AD-like male and female group). Measurements were performed in triplicate for each assay for each animal. Results show statistical significance between non-AD and AD-like degus (***p < 0.001) as well as males versus females (*p < 0.05) in AD-like group

**Fig. 3:**
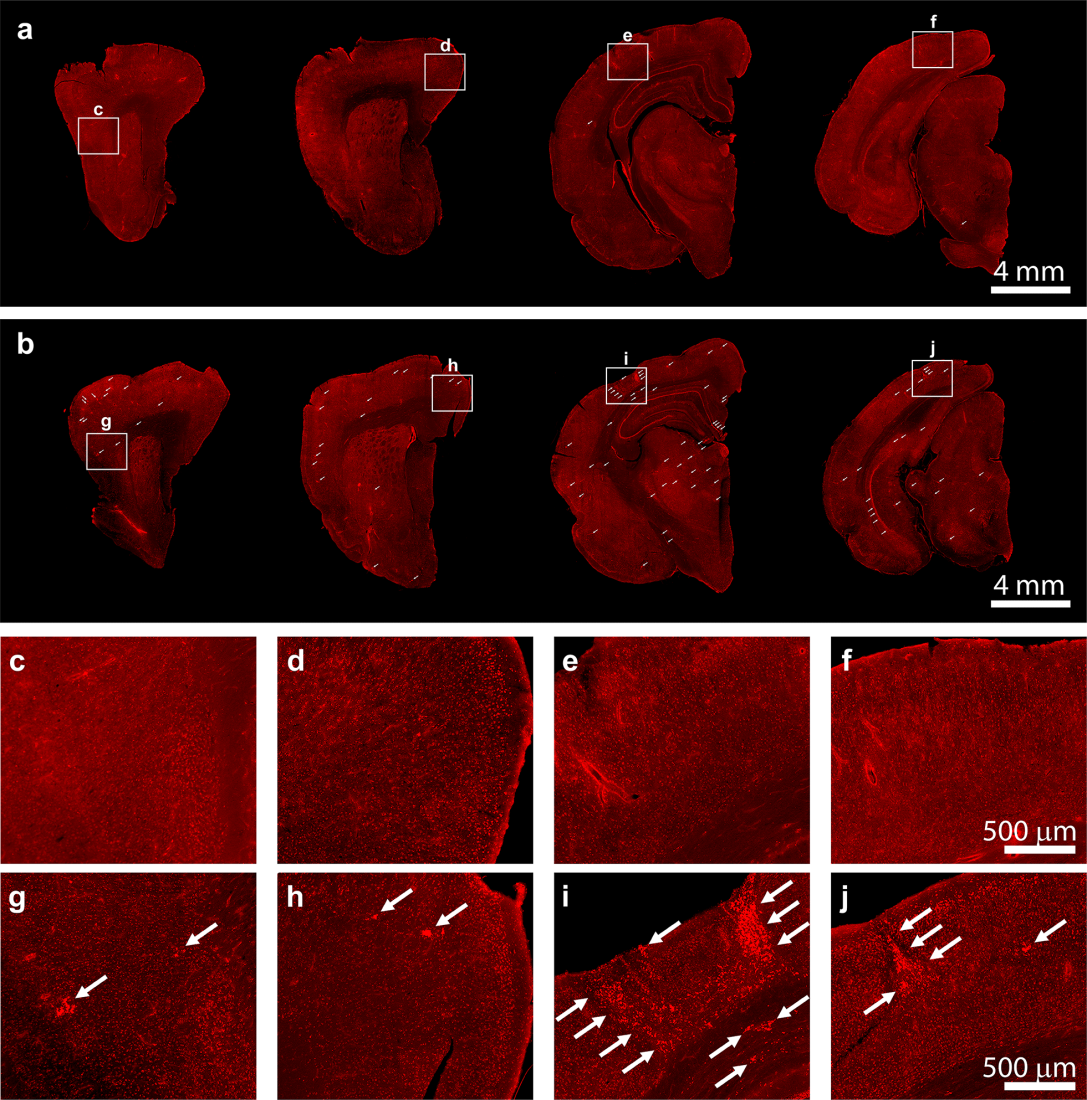
Enhanced Aβ deposits in AD-like degu brains. Immunofluorescence microscopic overviews of anterior-to-posterior (hemisphere) coronal sections of Non-AD (**a**) and AD-like (**b**) degu brains stained with 6E10 antibody (red). Zoomed-in views for Non-AD (**c**–**f**) and AD-like aged (**g**–**j**) degus are denoted with squares in the overviews. Arrows highlight selected Aβ plaques in different regions of the AD-like degu brain

**Fig. 4:**
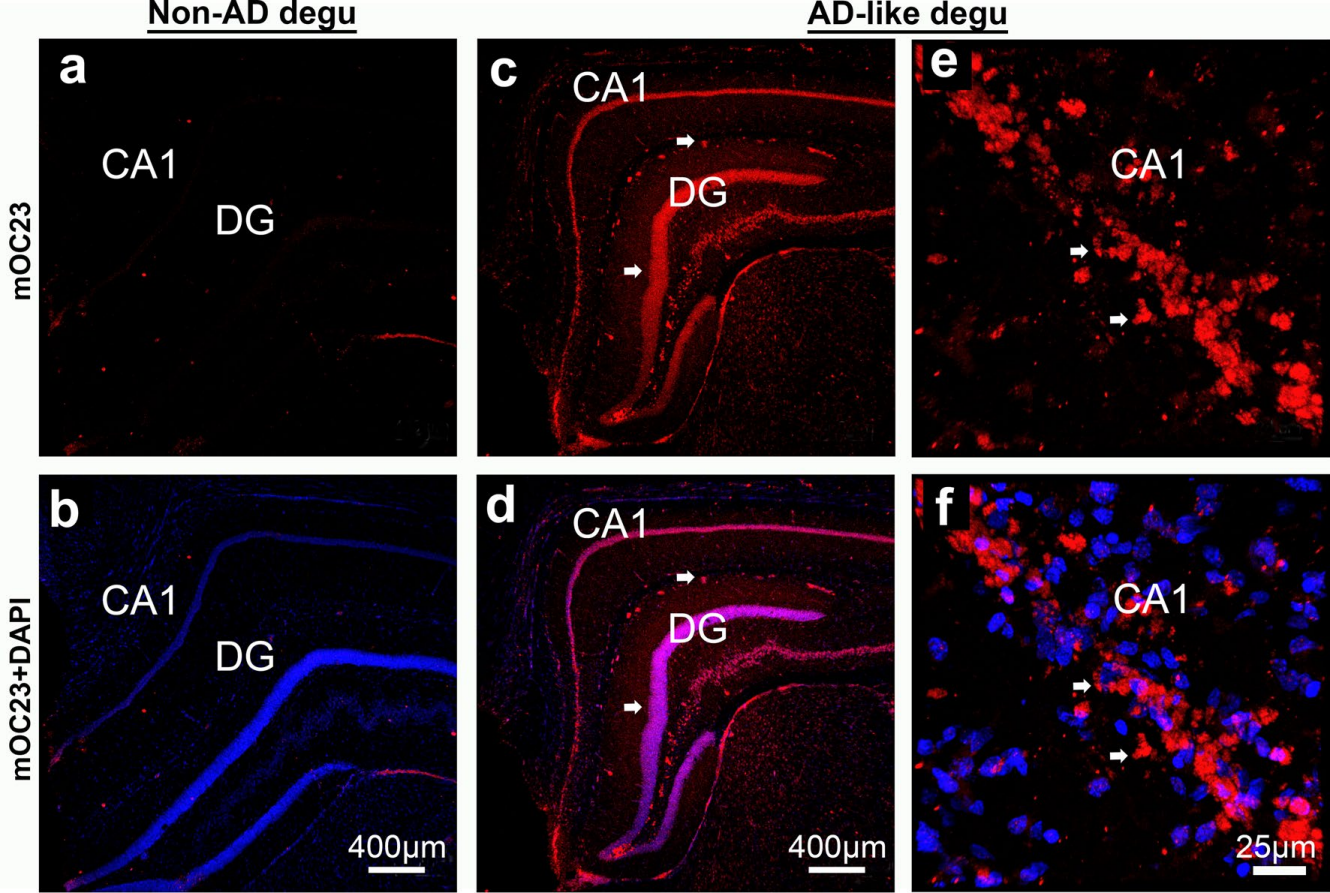
AD-like degu hippocampus shows oligomeric Aβ deposits compared to age matched controls. Immunofluorescence confocal microscopic images show enhanced immunoreactivity of Aβ oligomers stained by mOC23 rabbit monoclonal antibody (red) in hippocampus of coronal sections (right hemispheres) of non-AD (**a**, **b**) and AD-like (**c**, **d**) degus. A zoom-in view of a part of CA1 in (**c**) and (**d**) is shown in (**e**, **f**). Arrows indicate selected mOC23-positive Aβ deposits. Counterstained with DAPI in blue. Note that overlap in DAPI (blue) and mOC23 (red) signal results in magenta color

**Fig. 5:**
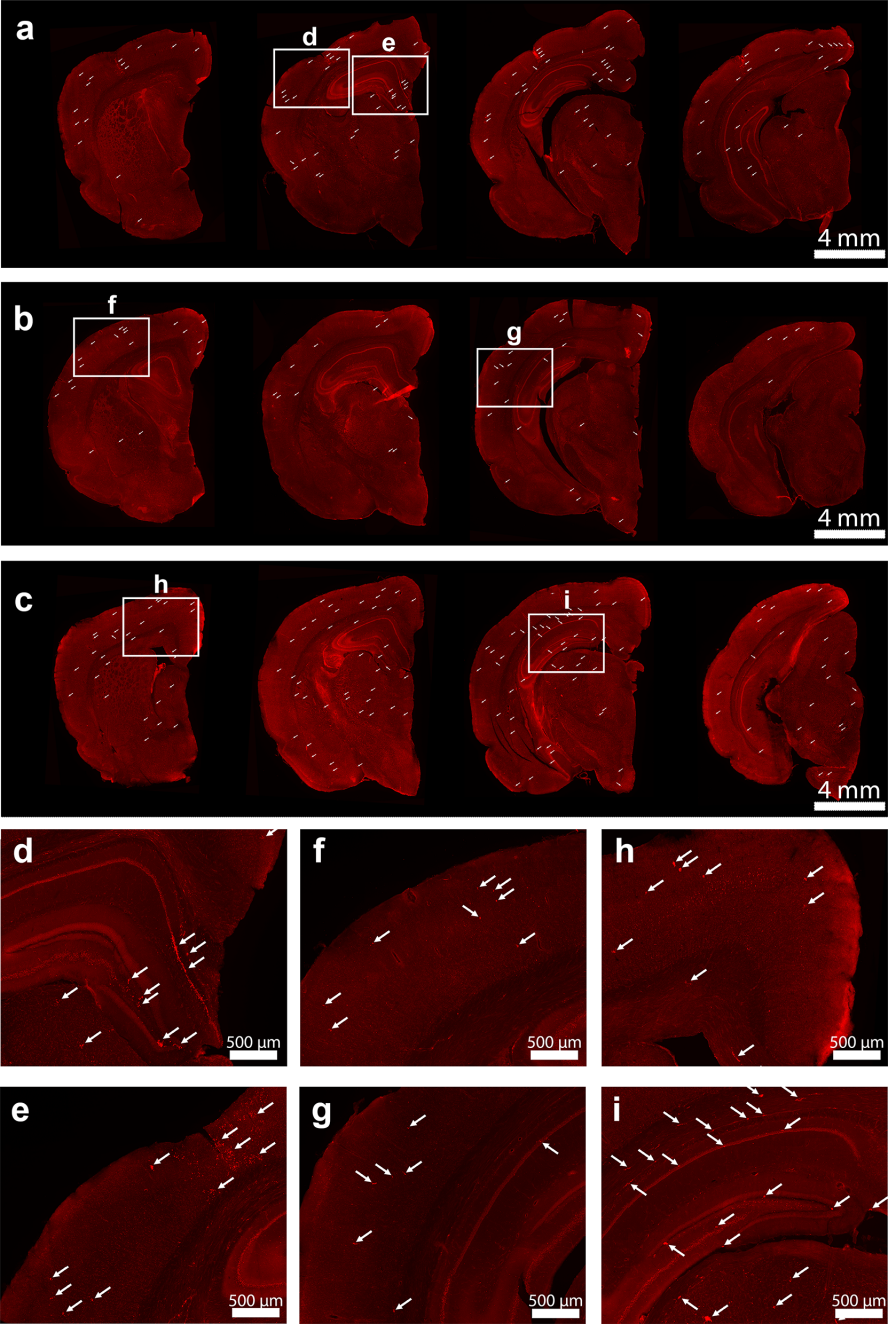
Oligomeric Aβ deposits in AD-like degu brain. **a**–**c** Immunofluorescence microscopic overviews of anterior-to-posterior coronal sections (from left to right) in three AD-like degus stained with mOC23 rabbit monoclonal antibody (red). **d**–**i** Zoom-in views of boxed areas in (**a**–**c**) show abundant oligomeric Aβ deposits in the AD-like degus. Selected oligomeric deposits denoted by arrows in both overviews and zoomed-in views

**Fig. 6:**
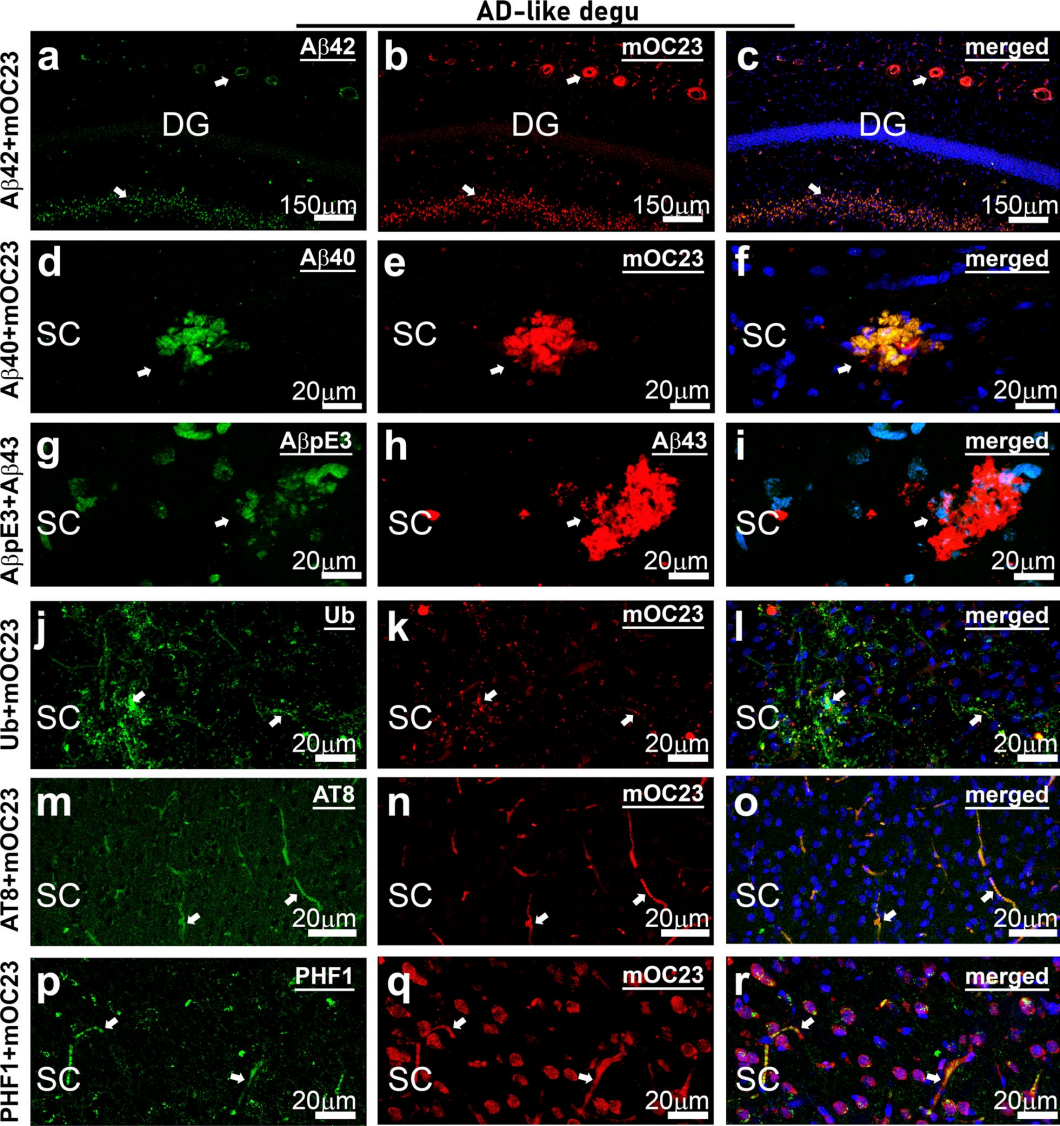
Aβ deposits in AD-like degu brain are immunoreactive to the common Alzheimer’s pathologic molecular markers. Immunofluorescence confocal micrographs double-labeling of mOC23 with Aβ42 (**a**–**c**) or Aβ40 (**d**–**f**), Aβ43 with pyroglutamate Aβ (AβpE3) (**g**–**i**), and mOC23 with ubiquitin (Ub) (**j**–**l**) or AT8 (**m**–**o**) or PHF1 (**p**–**r**) for phosphorylate tau. Prominent signal indicated by arrows in panels from the two left columns are overlapped in the corresponding merged panels **c**, **f**,**i**, **l**, **o**, **r** from the right column. Merged images show green and red channel with DAPI that stains nuclei blue. Merge of red and green results in yellow (**f**, **l**, **o**), and overlap of red with blue yields magenta (**c**, **f**,**i**, **l**, **o**, **r**). The staining pattern of mOC23 with ubiquitin or phosphorylated tau (AT8 and PHF1) exhibit neurite-like structures that are similar to those detected by 6E10 in Fig. 3e, suggesting that these are similar to neuritic plaques

**Fig. 7:**
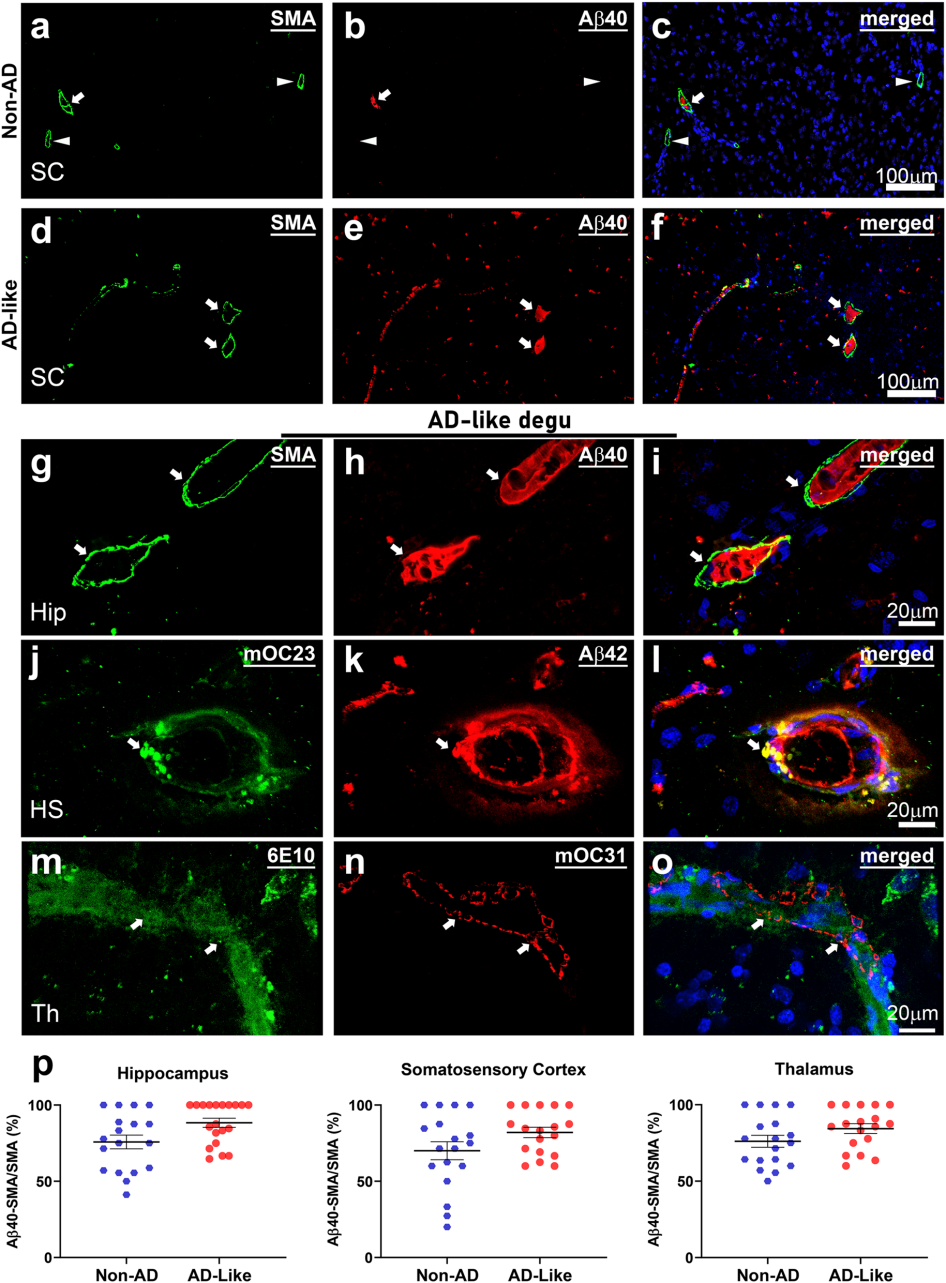
Cerebral amyloid angiopathy detected in the degu brain. **a**–**f** Immunofluorescence confocal microscopic images show dual immunofluorescence of smooth muscle a-actin (SMA, green), a molecular marker for small arteries and Aβ40 (red) and degu brains. Arrowheads indicate no Aβ40 in SMA-positive vessels a non-AD degu brain slice (**a**–**c**) when images merged with DAPI, which stains cell nuclei as blue, while arrows indicate SMA-stained vessels are associated with Aβ40 in non-AD and AD-like (**d**–**f**) degu brains. (**g**–**i**) A zoom-in view generated from a maximum intensity projection of Z-stack slices shows two SMA-stained vessels (green in **g**) enclosing Aβ40-positive deposits in the vascular wall (red in **h**) in merged images combined with DAPI (blue). Note: there is no autofluorescent bleed-through between the red and green channels, suggesting signal in each channel is specific. **j**–**o** Additional confocal microscopic images show partial colocalization of mOC23 (**j**) and Aβ42 (**k**) in **o**, arrow-pointed yellow parts) in hippocampus (Hip), and a spatial association of 6E10-stained deposits (green in **m**) and mOC31-positive vascular specific Aβ (red in **n**) in merged images **o** with DAPI (blue) in thalamus (Th) area. (**p**) Quantification is depicted as the percentage of both Aβ40- and SMA-positive vessels over all SMA-positive vascular structures in a 1.69 mm2 ROI’s (a 10x confocal image) and three non-overlapped ROI for each anatomical area were examined

**Fig. 8:**
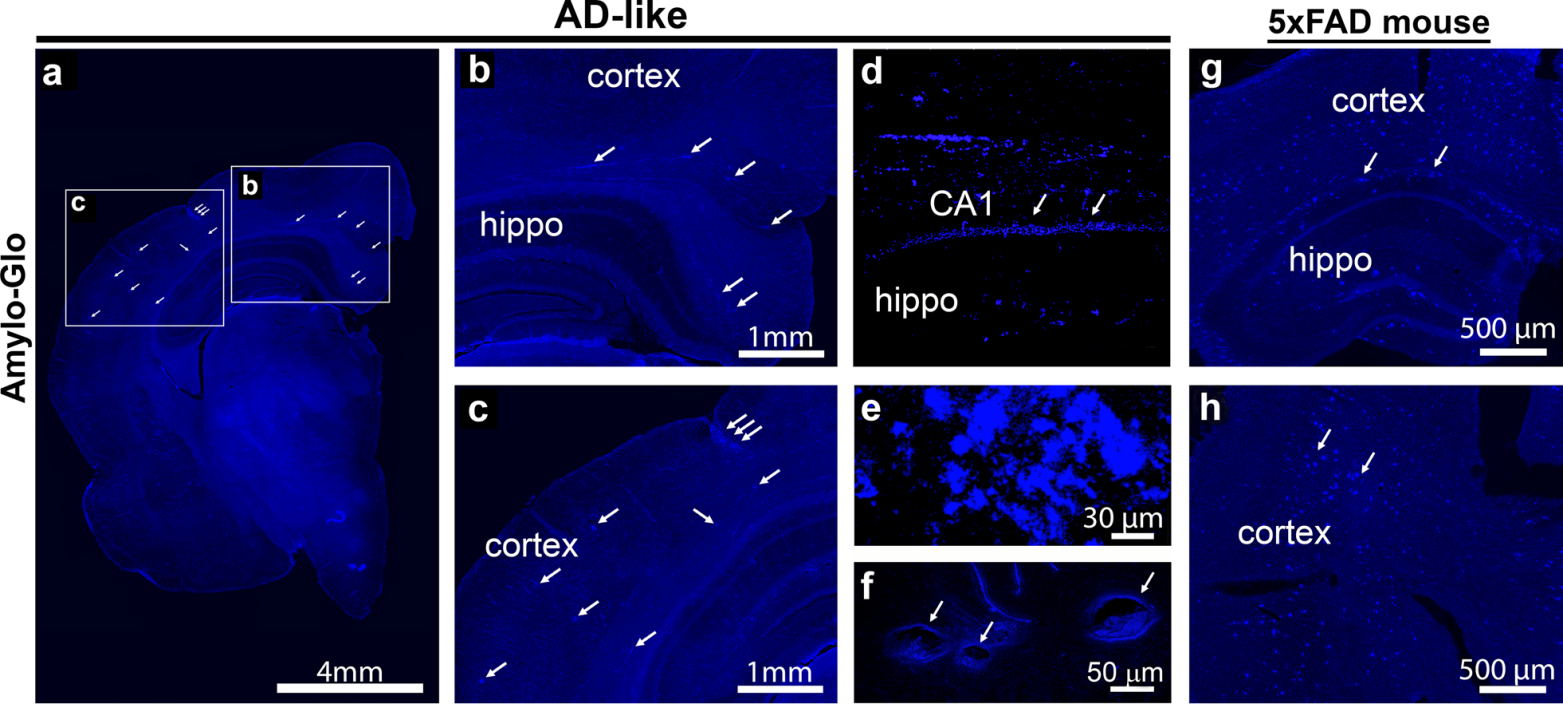
Abundant Aβ aggregates are stained by Amylo-Glo dye in AD-like degu brain. **a** Fluorescent microscopic images show Aβ plaques in a coronal section with hippocampus from an AD-like degu brain stained with Amylo-Glo fluorescent dye. **b**, **c **a higher magnified view of boxed areas in **a** shows Aβ plaques (arrows) in both hippocampus (hippo) and cortex areas. **d**–**f** A confocal microscopic view of hippocampal CA1 Aβ plaques (**d**), a large cluster of plaques in the cortical area (**e**), and several vascular structures within hippocampus. (**g, h**) A representative view of Amylo-Glo-stained coronal brain slices of 5xFAD mice shows Aβ plaques (arrows) in the cortex and hippocampus areas

To examine whether the AD-like aged degus with impaired burrowing performance show correlative AD-like brain pathologies, coronal sections containing brain areas known to contain neuropathological features in human AD and mouse models were stained with 6E10 and visualized by fluorescence confocal microscopy (Fig. 2). The monoclonal antibody (Mab) 6E10 is a widely used mouse monoclonal antibody directed against the N-terminal 1–16 amino acid residues of Aβ. As demonstrated by confocal micrographs in Fig. 3, very little Aβ immunoreactive staining is seen in both somatosensory cortex and hippocampal areas of Non-AD aged degu brains (Fig. 3a, b). In contrast, large numbers and high density of Aβ plaques are detected in the corresponding brain areas of aged degus that exhibit burrowing behavioral defects (Fig. 3c, d). Notably, prominent Aβ immunoreactive neuritic-plaque-like deposits are also found in the retrosplenial cortex of behaviorally impaired aged degus (Fig. 3e, f). A magnified view reveals cytoplasmic Aβ deposition in CA1 pyramidal neurons and large plaques in the cortex of these aged degus (Fig. 3g, h). The CA1 hippocampal region and retrosplenial area are strongly implicated as being important for object place memory [24–26]. These observations are corroborated by epifluorescence microscopic images (Fig. 2) that show detectable Aβ plaques in the cortex, hippocampus, and thalamus from AD-like degus with behavioral impairments (Fig. 2b, see also higher magnification images in panels g-j). In contrast, few putative Aβ plaques are seen in the corresponding brain level sections of the Non-AD group of aged degus (Fig. 2a & zoom-in panels c-f). Note that the appearance of spontaneously occurring Aβ pathology in the brains of aged degus that exhibit burrowing behavioral defects is distinguishable from the substantially more extensive plaques that are driven by familial mutations as measured in the aged 5xFAD mouse brain (Fig. 3i, j). Quantification of the total area of immunoreactive Aβ deposits in the degu anterior hippocampus and somatosensory cortex shows significantly higher levels of 6E10-positive deposits in AD-like aged degu brains compared to Non-AD aged degus (Fig. 3k). These observations are further corroborated by ELISA results measured in formic acid-extracted brain homogenates using Aβ40- and Aβ42-specific ELISA kits. Noticeably, both levels of Aβ40 and Aβ42 are significantly higher in the AD-like compared to the Non-AD aged degu brains, along with higher Aβ40 and Aβ42 levels in AD-like female degus compared to male AD-like degus (Fig. 3 L, m). Moreover, this Aβ pathology is supported by adjacent sections stained with mOC23, a rabbit Mab capable of recognizing oligomeric conformation Aβ aggregates, which we confirmed via dual immunofluorescence with Aβ40 and Aβ42 antibodies (Fig. 6a–f). While very little immunoreactive signal is found in Non-AD aged degu brains (Fig. 4a, b), robust mOC23-postive aggregates are seen in the hippocampus (Fig. 4c–f) and other brain areas of AD-like aged degus as demonstrated in the epifluorescence microscopic images (Fig. 5). Furthermore, this type of mOC23-stained deposits is immunoreactive for Aβ42 (Fig. 6a–c) and Aβ40 (Fig. 6d–f). Similar-looking Aβ plaques are also detected in adjacent slices with antibodies specific for Aβ43 and pyroglutamate AβpE3 (Fig. 6g–i), which are known to be the most toxic forms and major species of Aβ in the human AD brain [27, 28]. Importantly, double-labeling of mOC23 with ubiquitin (Fig. 6j–l), AT8 (Fig. 6m–o) or PHF1 (Fig. 6p–r) for phosphorylated tau demonstrate partial colocalization of mOC23 with ubiquitin or phosphorylated tau deposits, suggesting the presence of neuritic-plaque-like deposits. To further characterize the vascular deposition of Aβ in the degu brain as shown in Fig. 6a–c, adjacent coronal slices were double-stained for Aβ40 and smooth muscle α-actin (SMA). Immunofluorescence confocal micrographs identify Aβ40 immunoreactivity in most SMA-labeled small vessels in both Non-AD and AD-like degu brains (Fig. 7a–f). This is clearly shown in zoom-in views of the AD-like degu hippocampus (Fig. 7g–i). A similar staining pattern is also detected for mOC23 and Aβ42, and both are partially colocalized in association with vascular structures (Fig. 7j–l). Moreover, dual staining of 6E10 and mOC31, a rabbit Mab for conformation-specific vascular Aβ, followed by confocal microscopy confirms amyloid angiopathy (Fig. 7m–o). Quantification of Aß40 immunoreactive signal in α-SMA-positive vessels shows slightly higher levels of Ab deposits in hippocampus, somatosensory cortex, and thalamus of AD-like degus compared to the normal controls (Fig. 7p). In addition, the fibrillar form of Aβ is detected by Amylo-Glo staining of adjacent coronal brain slices collected from the brains of AD-like aged degus (Fig. 8). Amylo-Glo stains Aβ fibrils inside cells, plaques, and vascular structures in AD-like aged degu brains, visualized by epifluorescence (Fig. 8a–c) and confocal fluorescence microscopy (Fig. 8d–f). As a positive control, Amylo-Glo robustly stains Aβ fibrils in the 5xFAD mouse brain (Fig. 8g–h).

### Enhanced tau pathology in the brain of AD degus

**Fig. 9:**
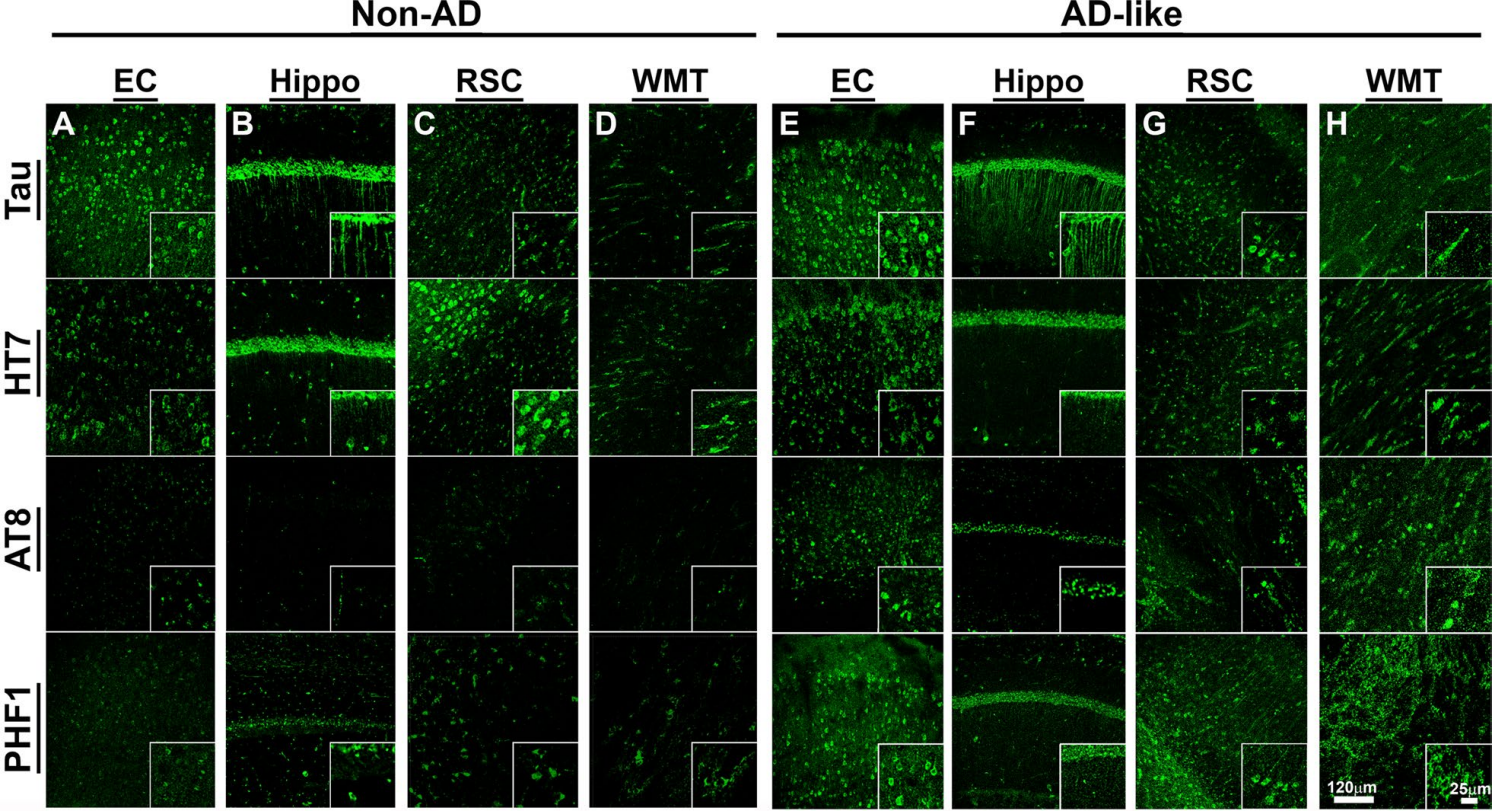
Enhanced tau pathology in brains of AD-like aged degus that exhibit behavioral burrowing defects. **a**–**h** Immunofluorescence confocal microscopic views of generic tau (Tau, HT7) and phosphorylated tau (AT8, PHF1) immunoreactivity in selected areas of coronal sections (right hemispheres) including hippocampus from a non-AD (**a**–**d**) and AD-like aged degus (**e**–**h**) demonstrate comparably equal fluorescent immunoreactivity of the total Tau protein by a rabbit monoclonal anti-Tau (159PPGQK163) and mouse HT7 monoclonal anti-Tau (306VQIVYK311 amyloid motif) antibodies, respectively. In contrast, greatly enhanced immunoreactivity for tauopathy markers AT8 and PHF1 was found in AD-like aged degu brains compared to the corresponding areas in Non-AD aged degus. The inset in each panel shows a high magnification view of the staining signal in individual cells. Entorhinal cortex (EC), hippocampus (Hippo), retrosplenial cortex (RSC), and white matter tracts (WMT)

Neurofibrillary tangles (NFT) resulting from abnormal accumulation of hyperphosphorylated tau proteins are another common pathologic hallmark in the human AD brain [29]. Degu tau is highly homologous to human tau protein [30]. As degu tau pathologies have not previously been carefully examined, we used two specific Mabs, HT7 (directed against the sequence motif 159PPGQK163 in human Tau40, corresponding to sequence PSGQK in degu tau) and A19560 (directed against the Tau amyloid motif 306VQIVYK311 that is identical in human and degu Tau proteins), respectively, to measure NFT-like structures in the degu brains. Strong Tau accumulation is found in multiple brain structures in AD-like aged degu brains, particularly in entorhinal cortex, hippocampus, retrosplenial cortex, and white matter tracts (WMT, Fig. 9, top two rows). When adjacent sections were stained with AT8 & PHF1 for hyperphosphorylated tau, immunofluorescence confocal microscopy also revealed prominent staining of whole brain sections in AD-like aged degus (Fig. 9, bottom two rows).

### Increased markers for neuroinflammation and neuronal loss in AD-like degu brain

**Fig. 10:**
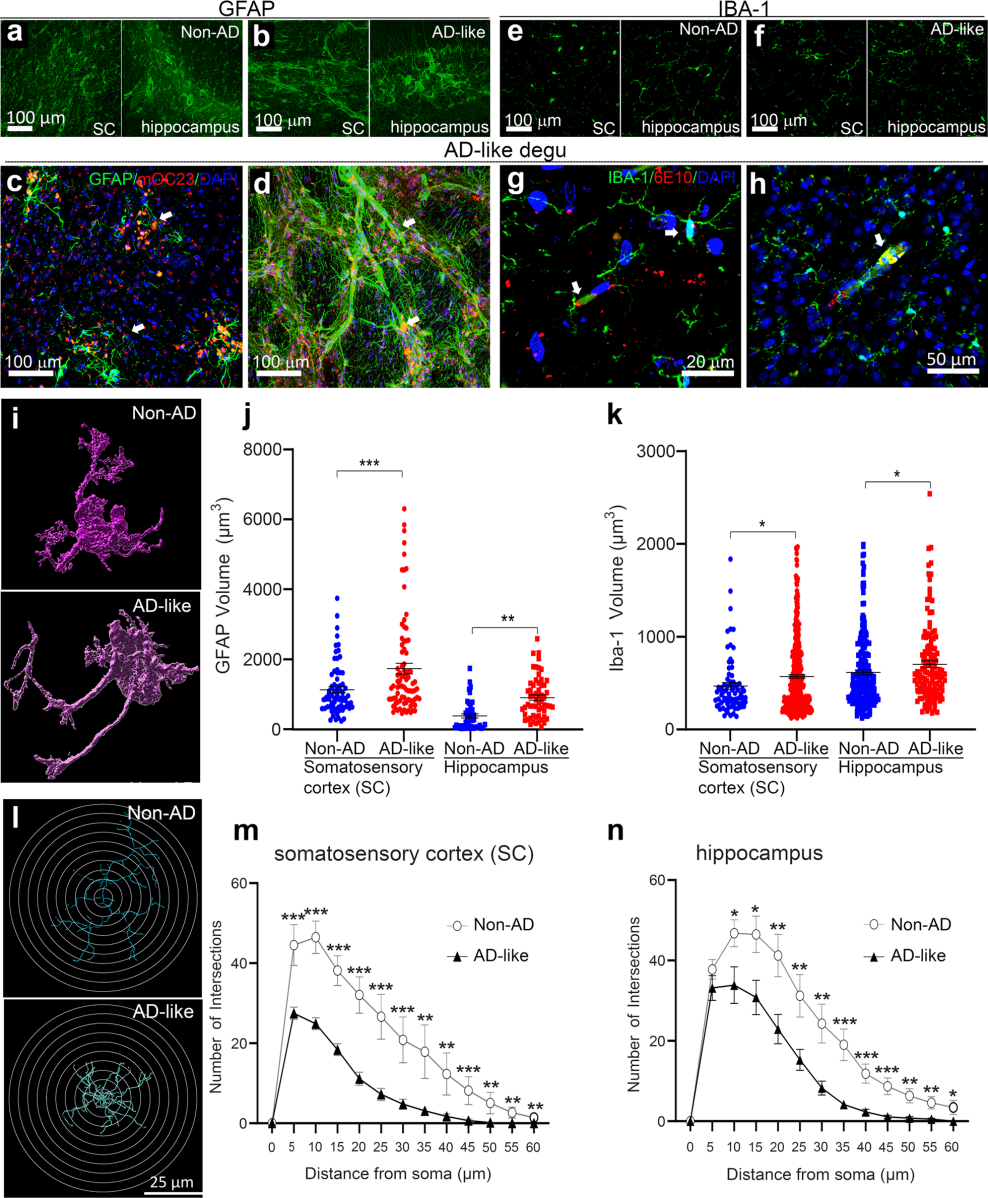
AD-like aged degu brain displays enhanced neuroinflammation relative to controls. **a**–**h** Reactive astrocytes and microglia measured by GFAP and IBA1 immunoreactivity, exhibit relatively lower intensity in control non-AD (**a**, **e**) compared to AD-like (**b**–**d**, **f**–**h**) aged degu coronal brain sections in confocal microscopic images. Higher magnified images show either colocalization of GFAP or IBA1 with Aβ aggregates stained by mOC23 (red) or 6E10 (red) in plaques or accumulation surrounding plaques (**c**, **g**) and vasculatures (**d**, ** h**) as indicated by arrows in AD-like degu brains. **i** Imaris-based 3D reconstructions of representative GFAP-positive astrocytes from Non-AD and AD-like aged degu brain cortex. **j**, **k** Quantification of GFAP- (**j**) or IBA1-positive (**k**) cells in somatosensory cortex (SSC) and hippocampus shows a significant increase in both GFAP and IBA1 volumes in AD-like compared to Non-AD aged degu brains. (**l-n**) Sholl analysis conducted using Imaris in the filament reconstruction mode shows two representative IBA1-positive microglia from Non-AD and AD-like aged degu brain cortex (**i**), respectively; mean distribution plots of Sholl intersection numbers versus the distance from the microglial soma demonstrate a significant decrease in the total number of intersections of microglial cells in both in cortex (somatosensory cortex) (**m**) and hippocampus (**n**) in AD-like aged degu brains compared to age-matched Non-AD degus. The scale bar represents 100 μm (**a**, **b**, **e**, **f**) and 40 μm (**c**, **d**, **g**, ** h**). *p* < 0.01*, 0.001**, or 0.0001***

**Fig. 11:**
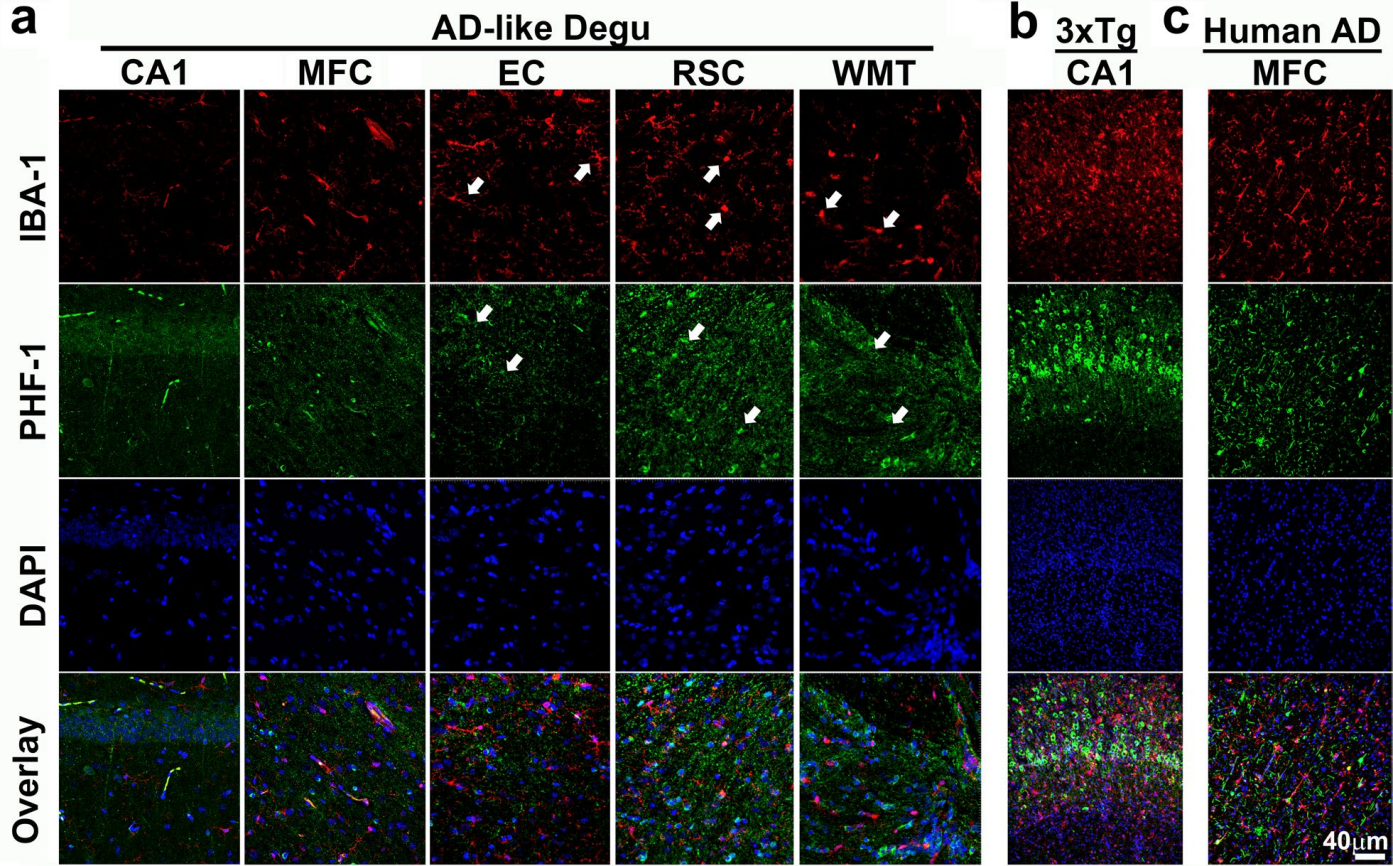
AD-like degus exhibit similar distribution patterns of tau pathology and microglia activation as those seen in the 3xTg mouse model and human AD. (**a-c**) Confocal immunofluorescence micrographs show microglial cells labeled by IBA-1 (red, arrows) and neurofibrillary-tangle-like structures stained with PHF-1 antibody (green, arrows) in the specific regions including hippocampal CA1, MFC (medial frontal cortex), entorhinal cortex (EC), retrosplenial cortex (RSC), and white matter tracts (WMT), areas in the AD-like degu brain (**a**), and those in hippocampal CA1 in 3xTg-AD mouse (**b**), and in medial prefrontal cortex (MFC) from a postmortem human AD brain (**c**). Cell nuclei are counterstained with DAPI as blue

Recent evidence shows that neuroinflammation contributes to AD pathogenesis [31, 32]. We tested whether increased expression of inflammatory cytokines and cellular markers for neuroinflammation are detected in aged degu brains with AD-like behavioral burrowing impairments as compared with age-matched control degus without behavioral impairments [14]. To measure neuroinflammatory markers in the degu brains in this study, coronal sections containing the hippocampus were stained for GFAP (astrocyte marker) and IBA1 (microglia). Fluorescence confocal microscopic images show qualitatively stronger immunoreactivity for both GFAP and IBA1 in the cortex and hippocampus areas of AD-like aged degu brains compared to the age-matched Non-AD degus (Fig. 10a, b, e, f). Recapitulating the neuropathological features of human AD, co-immunostaining of GFAP and IBA1 reveals direct spatial associations of GFAP-positive astrocytes and IBA1-immunoreactive microglia with Aβ aggregates, with some of the activated astrocytes and microglia entangled with vascular structures harboring Aβ aggregates (Fig. 10c, d, g, h) indicating prominent angiopathies. 3-D reconstruction images reveal the morphological differences between cortical astrocytes in Non-AD and AD-like aged degus (Fig. 10i), and quantification shows increases in cell volumes for both GFAP-labeled astrocytes and IBA-1-positive microglia in both somatosensory cortex and hippocampus of the AD-like degus compared to age-matched Non-AD degus (Fig. 10j, k). Furthermore, Sholl analysis was conducted to measure the number of cross-over events of microglial processes through 5 μm radially-increased concentric rings and nodes as shown in the illustrations (Fig. 10L). The Sholl quantification plots clearly depict a significant decrease in the branching numbers of microglia in both cortical and hippocampal areas of the AD-like aged degus compared to the Non-AD aged degus (Fig. 10m, n). The morphological features of astrocytes and microglia in AD-like aged degu brains indicate enhanced neuroinflammation in AD-like degu brains relative to age matched Non-AD degus. In contrast to the colocalization of most IBA-1 staining with Aβ plaques, little IBA-1 staining colocalization is seen with paired helical filaments as measured using PHF-1 staining in the brains of AD-like aged degus, and their distributions are similar to those observed in the CA1 region of 3xTg AD transgenic mouse brains and the medial frontal cortex of postmortem human AD brains (Fig. 11).

**Fig. 12:**
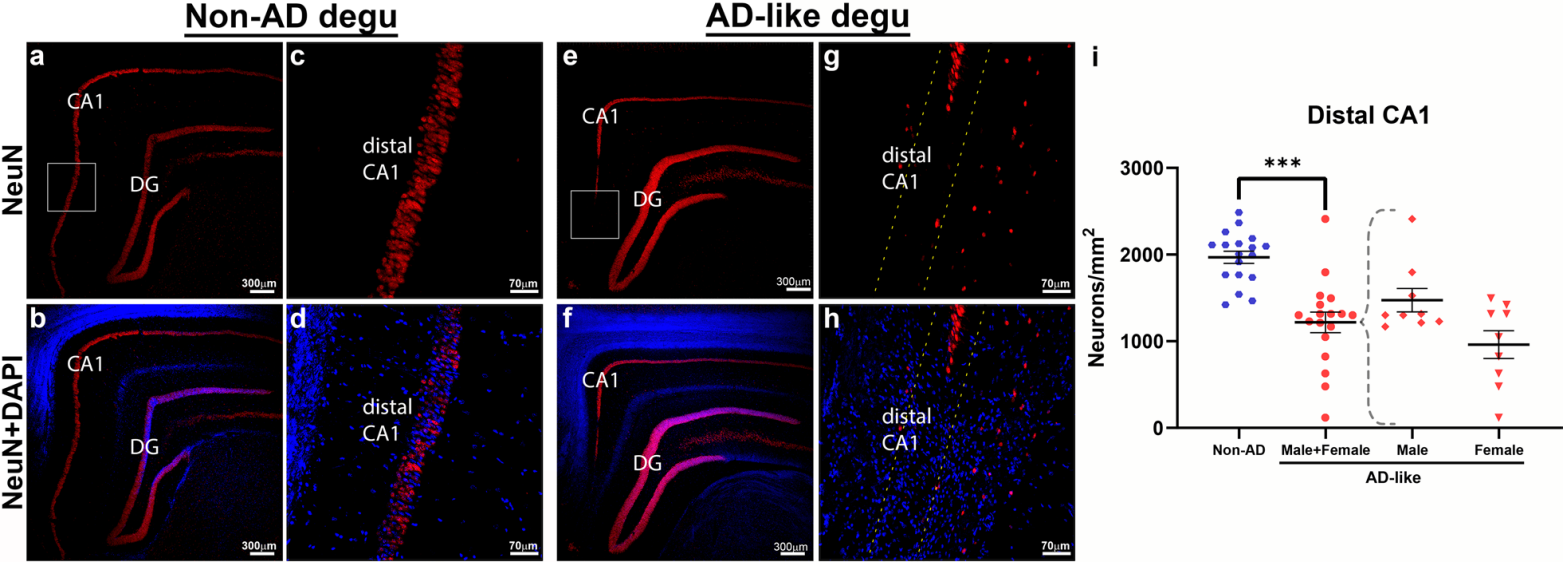
Significant neuronal loss in hippocampus of AD-like aged degu brains. Confocal microscopic images demonstrate the distribution of neurons labeled by NeuN immunoreactivity (red, a specific marker for neurons) in the hippocampus. (**a, b**) A low-magnification image shows NeuN-labeled neurons in CA1 and dentate gyrus (DG) regions in a Non-AD degu coronal brain section. **c**, **d** A higher magnified view of the boxed area in (**a**) shows NeuN-labeled pyramidal neurons in distal CA1. **e, f** An overview image shows a notable loss of NeuN signal in the distal CA1 region in an AD-like aged degu brain. **g**, **h** A higher magnified view of the boxed area in **g** highlights NeuN signal missing in the distal CA1 (the area between two dotted lines). Cell nuclei are counterstained by DAPI (blue) in **b**, **d**, **f**, **h**. Note that overlap in DAPI (blue) and NeuN (red) signal results in magenta color. (**i**) Quantification of NeuN-positive cells in CA1 shows significantly fewer neurons in AD-like degu brain compared to Non-AD control (*p* = 0.0006714) and a trend towards greater loss in AD-like female degus compares to AD-like males (*p* 0.076432). Error bars represent SEM; ***p < 0.001, n = 6 degus in AD-like and Non-AD groups, n = 3 for male AD-like and female AD-like groups

Neurodegeneration is one of the main neuropathological features in the human AD brain and accounts for the significant brain atrophy seen in these patients [33, 34]. Although most transgenic animal models of AD demonstrate Aβ- and neuroinflammation-related pathology in the brain, many of them fail to recapitulate the neurodegeneration and neuronal loss seen in human AD brains [35]. To examine whether there is neuronal loss in the brains of degus with AD-like behavioral impairments, brain coronal sections were specifically labeled and quantified using neuron-specific nuclear NeuN immunofluorescence followed by confocal microscopy. We focused our investigation on hippocampal CA1 because cell loss in this brain region is commonly seen in human AD patients. As shown in confocal micrographs in Fig. 12, NeuN-immunoreactive neurons are clearly seen in the entire CA1 pyramidal cell layer of control Non-AD aged degu hippocampus (Fig. 12a–d). In contrast, NeuN signal is not detected in a substantial part of the distal CA1 pyramidal layer of AD-like aged degus (Fig. 12e–h). This significant loss of CA1 hippocampal neuronal cells is further confirmed by cell counting (Fig. 12i).

**Fig. 13:**
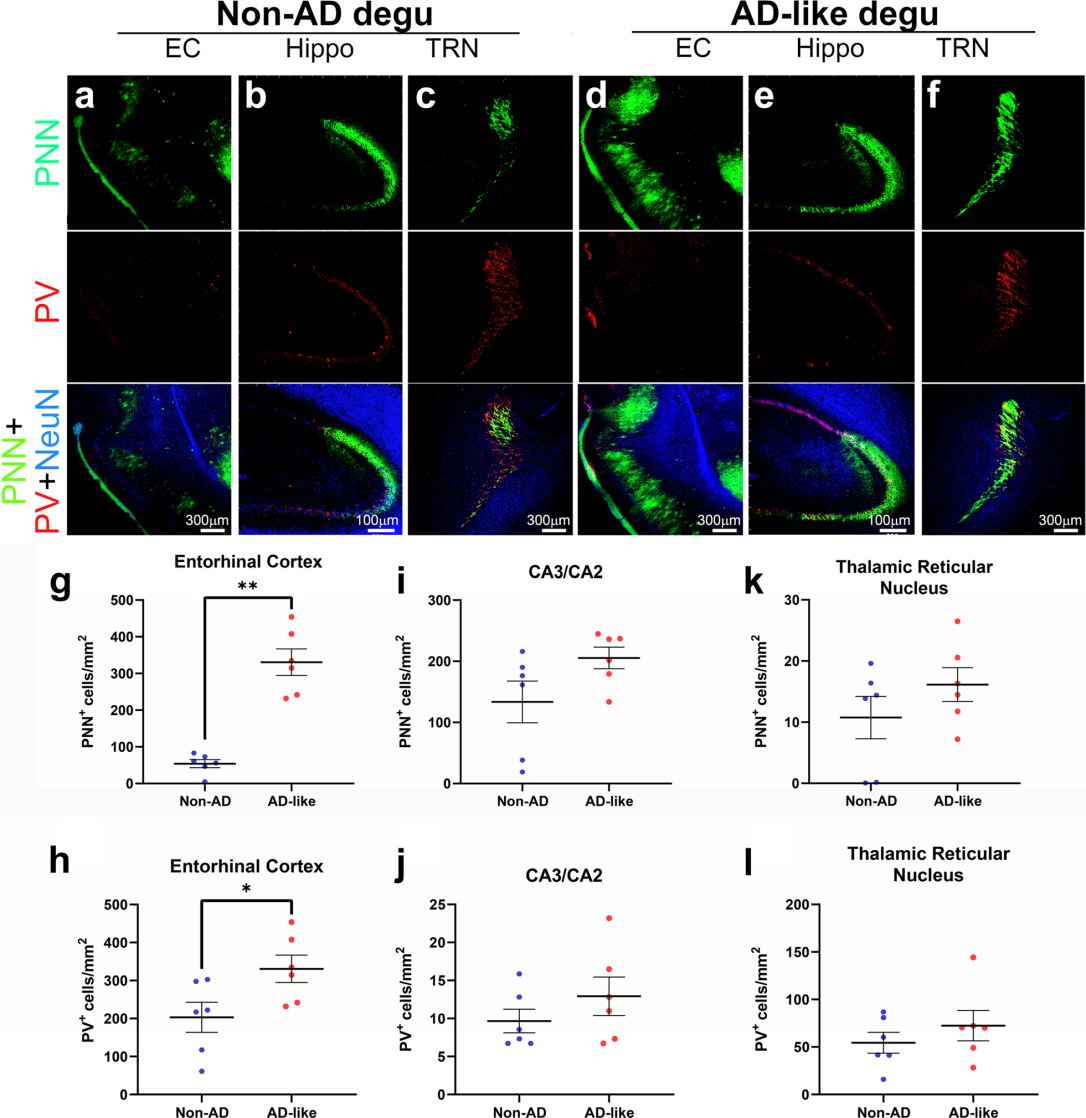
Increased perineuronal nets signal coupled with an increase of PV-positive interneurons in the AD-like aged degu brain. Confocal microscopic views of degu coronal brain sections are aligned as indicated and show focused views of perineuronal nets (PNN) (green), parvalbumin (PV) interneurons (red), and neuronal nuclei (NeuN, blue) in entorhinal cortex (EC, **a** and **d**), hippocampal CA2 and CA3 (Hippo, **b** and **e**), and thalamic reticular nucleus (TRN, **c** and **f**). **g**–**l** Quantification shows significantly increased counts of PNN(+)-neurons per mm^2^ (**g**) and PV(+)-neurons per mm^2^ (**h**) in EC (*p* = 0.0022 and  *p* 0.0260, respectively) in AD-like degu brains compared to Non-AD controls, along with similar trends for hippocampal CA3/CA2 (*p*  = 0.0931 for PNN; *p* = 0.3636 for PV) and TRN (*p* = 0.3939 for PNN; *p* = 0.5887 for PV). Error bars represent SEM; *p< 0.05, **p < 0.01, n = 6 degus in AD-like and Non-AD groups, n = 3 for male AD-like and female AD-like groups

Evidence suggests that signal intensity and the number of parvalbumin (PV)-expressing interneurons and perineuronal nets (PNN) is reduced in a variety of brain disorders including AD [36, 37]. We measured immunoreactivity of both PV and PNN in degu brains. As shown in Fig. 13, a slight increase in the intensity and the number of both PNN- and PV-positive cells is detected in the entorhinal cortex of AD-like aged degus compared to Non-AD aged degus (Fig. 13a, d). Note that due to the absence of PNN signal in hippocampal CA1, PNN staining in the degu hippocampus better resembles that of the rat hippocampus than the mouse hippocampus [38]. Changes in PNN and PV immunoreactivity is not evident in other areas such as the hippocampus and the thalamic reticular nucleus (Fig. 13c, f). These are confirmed by counting of PNN- and PV-immunoreactive cells (Fig. 13g–l).

### Increased c-Fos activation in AD-like aged degu brain

**Fig. 14:**
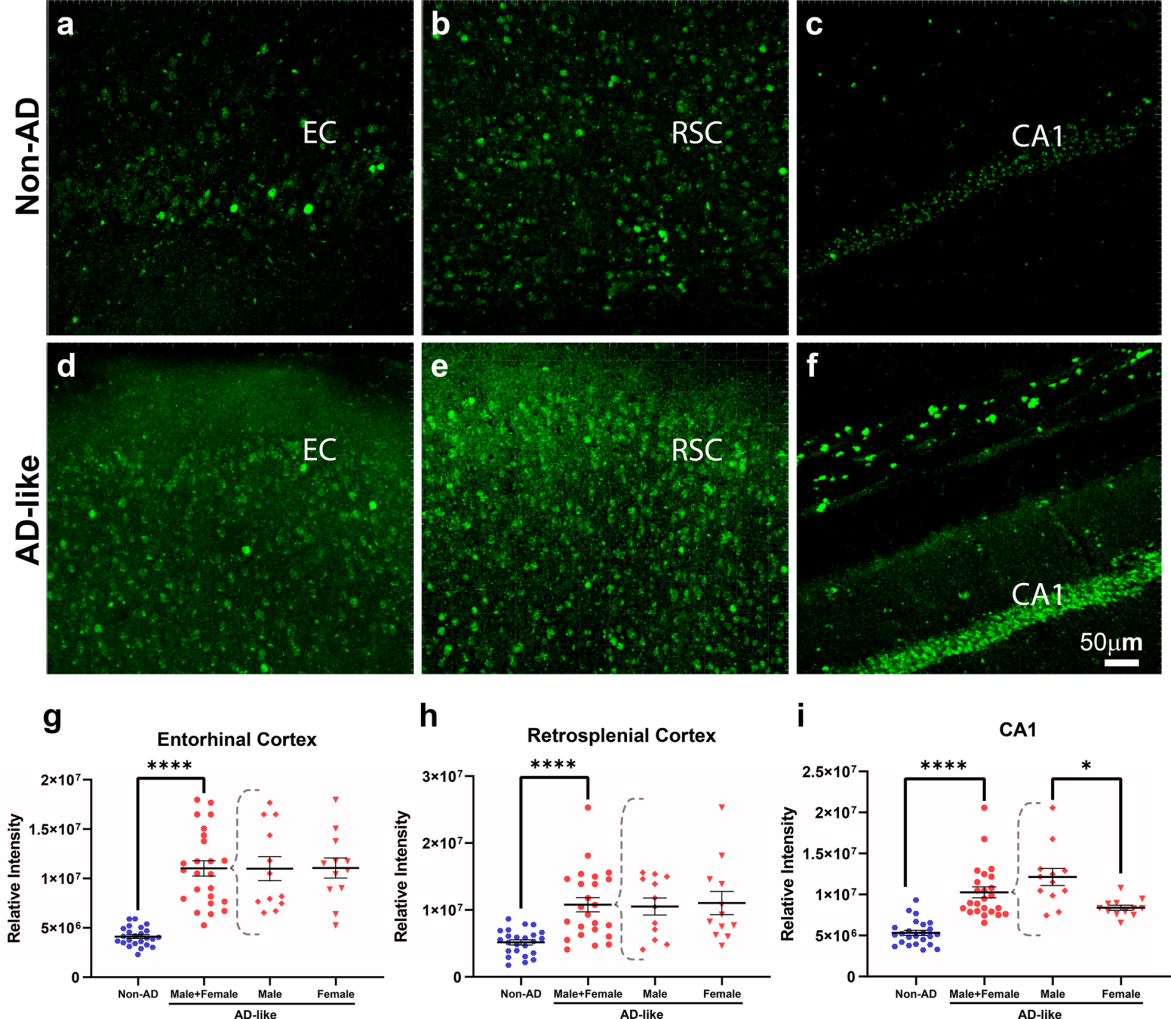
Enhanced c-Fos immunoreactivity in AD-like degus. (**a**–**f**) Immunofluorescence confocal microscopy of degu coronal brain sections for c-Fos (green) active neuron comparison between non-AD (top row) and AD-like aged degus (bottom row) in entorhinal cortex (EC, **a**, **d**), retrosplenial cortex (RSC, **b**, **e**) and hippocampal CA1 (**c**), **f** regions. **g**, **h**, **i** A significant increase in c-Fos signal measured as relative intensity value is found in entorhinal cortex (EC, **g**, *p* = 2.8 × 10^− 6^), retrosplenial cortex (RSC, **h**, p = 2.7 × 10^− 5^) and CA1 hippocampus (**i**, *p* = 7.9 × 10^− 5^) of AD-like degu brains compared to the Non-AD controls. A significant increase in c-Fos signal is also seen in male AD-like degus compared to Non-AD controls (*p* = 0.021). Error bars represent SEM; *p < 0.05, **p < 0.01, ***p < 0.001, ****p < 0.0001, n = 6 degus in AD-like and Non-AD groups, n = 3 for male AD-like and female AD-like groups

The protein expression of the immediate early gene *c-fos* is a marker of cellular activity in neurons. A number of reports indicate neuronal hyperactivity in human AD and AD mouse models [39]. In this regard, we examined c-Fos immunoreactivity in the degu brains. Compared to Non-AD aged degu brains, a significant increase in c-Fos signal is detected in the entorhinal cortex, retrosplenial cortex and hippocampal CA1 areas of the AD-like aged degu brain (Fig. 14). The AD-like aged degus show increased c-Fos signal in all measured brain regions, suggesting broad neuronal activation and underlying hyperactive circuitry in the aged AD-like degu.

## Discussion

In the present study, our comprehensive characterization of AD-like neuropathological features based on degus parsed by the presence or absence of behavioral burrowing performance impairments show that outbred degus are a natural model of sporadic human AD. As we are interested in capturing a model of the spontaneous appearance of AD in humans, we reason that like humans, not all aged degus will exhibit AD-like phenotypes. As AD is outwardly characterized by cognitive impairments, we first screened degus by assaying the presence or absence of cognitive deficits using an ethologically relevant burrowing behavior paradigm. Burrowing performance requires hippocampus function and is a more relevant behavior assay for assessing cognitive status in burrowing rodents like degus [40–42]. Our data show that about 1/3 of outbred aged degus show burrowing behavioral deficits. This is critical as AD does not occur in all aged humans. Degus demonstrating the lowest 25% and top 75% burrowing performance are grouped as “AD-like” and “Non-AD” degus, respectively. We compared correlative neuropathology between these two groups; the “AD-like” degus with impaired burrowing performance (no burrowing during the test period) and the age-matched “Non-AD” control degus with intact burrowing performance (> 75% burrowing of pellets during the test period). Our strategy is to purposefully exclude the degus with intermediate burrowing performance as such animals are more likely to have intermediate neuropathological features and thus potentially introduce noise in the data. We find significantly higher amounts of Aβ plaques as well as cytoplasmic Aβ deposits in the brains of degus with AD-like cognitive deficits compared to Non-AD controls. Like human AD, Aβ plaques in “AD-like” degus contain various forms of pathogenic Aβ variants immunoreactive to pyroglutamate (pE)-modified AβpE3, Aβ43, Aβ42, and Aβ40 isoforms in addition to conformation specific aggregates (oligomeric and fibrillar). These are widely distributed throughout the brain in areas including the cortex, hippocampus and thalamus - all brain regions with pronounced Aβ plaques in late stage human AD [4]. Also similar to human AD, these Alzheimer’s pathologic changes exhibit notable sex disparity, in which female degu brains show significantly more severe lesions including Aβ deposition and neuroinflammation than males. One drawback of many transgenic AD mouse models is that often such models do not reliably present pathological NFT-like structures [35, 43] that are robustly observed in the brains of human AD patients. In AD-like degus, increased Aβ deposits parallel the distribution of NFT-like structures detected by AT8 and PHF1 immunoreactivity. Importantly in AD-like aged degus, measurable increases in tauopathy are detected in the areas of white matter tract, retrosplenial cortex, and thalamic reticular nucleus, which are known to be vulnerable in human prodromal AD [44–47]. These neuropathological changes are also accompanied by measurable markers of neuroinflammation and perivascular pathology in AD-like degus, shown by significant changes in morphological features of astrocytes and microglia, respectively. These findings clearly demonstrate multiple spontaneous AD neuropathologies in the brain of degus with cognitive deficits and are consistent with spontaneous AD in humans that can be correlatively linked to behavioral impairment [48].

It is important to note that AD-like degu hippocampal sections clearly show loss of CA1 excitatory neurons as compared with non-AD like degu sections. This resembles the significant feature of neuronal loss in human AD brain (robust neurodegeneration), which is hard to recapitulate in most mouse AD models. Given that balanced cortical excitation and inhibition is required for normal neural circuit operations, we further examine the alterations of PV inhibitory neurons in AD-like degu brains. While reduction in the numbers of PV-expressing neurons has been measured in both brains of human AD patients and transgenic AD mouse models [49–51], an increase in the number of PV-positive GABAergic interneurons in hippocampus was associated with cognitive impairments in the dystrophic mdx mouse [52]. Here we find slightly more PV-expressing cells in the entorhinal cortex of AD-like aged degu brains versus Non-AD aged degus. PV-expressing GABAergic interneurons are normally ensheathed by perineuronal nets (PNN), a distinct extracellular matrix structure containing chondroitin sulfate proteoglycans in the brain [36]. Consistent with the increase in PV-expressing cells, the number of WFA-labeled PNN-positive cells is also increased in EC area of AD-like degus relative to the Non-AD. Interestingly, further studies are warranted to determine whether these alterations along with increased expression of c-Fos protein in the AD-like degus brain directly contribute to the increased vulnerability of neuronal cells and/or the pathogenesis of cognitive impairment in AD-like degus.

Our present study was motivated in part to settle earlier debates of whether degus can be a useful natural model of AD as particularly highlighted by the recent NIH RFA (RFA-AG-21-003). On retrospective reflection, we suspect that inconsistent findings between different studies may have been due to comparing neuropathology results from laboratory in-bred colonies versus more genetically diverse outbred degus, relatively low statistical power for sample size and the absence of behavioral screening [8, 10–12, 53]. Multiple gene alleles correlate with AD risk [54, 55]. Several mutations in APP, PSEN1, PSEN2, or ApoE4 alleles have been linked to the susceptibility of familial AD and early onset of the disease [56]. Since only a subset of degus show AD-like phenotypes, our data support the potential importance of wild-type outbred genetic backgrounds for the development of AD-like neuropathology and the potential power for genetic screening of mutations in familial AD genes. We hold that genetically diverse outbred populations will be more reliable for evaluating spontaneous AD-like features, and better reflect AD-associated allelic distributions that are of interest in human AD. A further complication of the earlier degus findings is that the ages tested are inconsistent between studies. Studies using younger animals (2–3 years old) do not show any AD-like effects, while more reliable AD-like effects have been observed in older degus (5–6 years old) [10, 57]. Given that the degu’s lifespan ranges from 5 to 8 years [12, 58], the age of around 5 years for the degus examined in our study may correspond to intermediate or advanced ages in human beings. Other likely sources of variance include low sample size and lack of behavioral screening that distinguishes normal versus impaired animals.

Outbred aged degus possessing both behavioral and neuropathological characteristics that resemble human AD pathologies, have clear advantages over common rodent models (mice and rats) for studying AD. Long-lived degus might reflect the importance of a long lifespan for AD neuropathology to manifest. When compared to other long-lived natural AD models, such as canines and non-human primates, degus have shorter lifespan, smaller size (similar to a rat), diurnal biological rhythm, and docile/social temperament, which make them a more cost effective and practical model for the laboratory setting. Further, a portion of the outbred degu population naturally develops additional conditions like type-2 diabetes, macular degeneration, and atherosclerosis with age, which provides an avenue to investigate AD comorbidities in the degu [59].

Taken together, our results show spontaneous AD-like correlative phenotypes in cognitive performance and neuropathology in aged outbred degus. This supports that aged degus are a useful and practical model of natural sporadic AD.
